# Design, Synthesis, and Antitumor Evaluation of Novel Mono-Carbonyl Curcumin Analogs in Hepatocellular Carcinoma Cell

**DOI:** 10.3390/ph15080950

**Published:** 2022-07-30

**Authors:** Pan Yu, Weiya Cao, Linguo Zhao, Qing Han, Shilong Yang, Kepeng Yang, Xiaolei Pan, Qianyun Wang, Yuan Wang

**Affiliations:** 1 College of Medicine, Anhui University of Science and Technology, Huainan 232001, China; 2020035@aust.edu.cn (P.Y.); ykp_2001@163.com (K.Y.); panxiaolei0622@126.com (X.P.); wqy147_369@163.com (Q.W.); 13605344787@139.com (Y.W.); 2 College of Chemical Engineering, Nanjing Forestry University, Nanjing 210037, China; yshl6072@njfu.edu.cn; 3 College of Science, China Agricultural University, Beijing 100193, China; hanqing1120@hotmail.com

**Keywords:** curcumin analogs, antitumor activity, cell apoptosis, AKT inhibition, molecular dynamics simulations

## Abstract

Curcumin is a polyphenolic natural product that has promising anticancer properties. However, its clinical utility is limited by its chemical instability and poor metabolic properties. In this paper, a series of new curcumin analogs were synthesized and found to be potent antiproliferative agents against the HepG2 cell line by MTT assay. In general, Group B with single ketone and group C with chalcone were markedly more cytotoxic than group A with diketone. Compound **B5** was found as the most potent analog (IC_50_ = 11.33 μM) compared to curcumin (IC_50_ = 32.83 μM) and the mechanism of its cytotoxicity was investigated. The result of the wound healing assay indicated **B5** strong potential to suppress HepG2 cell migration in a dose- and time-dependent manner. Subsequent assays (including JC-1 staining, Bcl-2, and caspase 3 protein levels by Western blotting) confirmed that **B5** exposure induced apoptosis in HepG2 cells. Curcumin-induced comprehensive transcriptomes profile, Western blotting, molecular docking, and molecular dynamics analysis showed that the mechanism may relate to the regulation of cellular metabolic process and the expression of AKT protein. Taken together, we could conclude that curcumin and its analogs induced HepG2 cell proliferation, migration, and apoptosis via AKT signaling pathway and the mitochondrial death pathway. This study could lay the foundation for optimizing curcumin and provide valuable information for finding novel anti-HCC drugs.

## 1. Introduction

Curcumin (1,7-bis(4-hydroxy-3-methoxyphenyl)-1,6-heptadiene-3,5-dione), as a natural product isolated from the rhizome of *Curcuma longa*, is mainly used for coloring of intestinal products, canned products, sauces, and marinated products in food production. Meanwhile, it has been found exhibiting various bioactivities, especially antitumor activity [1]. Without significant toxic, genotoxic, and teratogenic properties, curcumin was classified as a third-generation chemo-preventive agent for cancer by National Cancer Institute (NCI) [2]. Studies revealed that curcumin can inhibit cell proliferation and promote apoptosis of cancer cells through decreasing Bcl-2 protein expression [3,4], decreasing the phospho-AKT protein expression [5,6], and then increasing cleaved-caspase-3 activity [7,8]. Unfortunately, many clinical trials in various human cancer therapies of curcumin remained limited due to its relatively low potency and poor bioavailability [9]. Therefore, there has been considerable recent interest in developing curcumin analogs with highly active and clinically promising results.

The poor bioavailability of curcumin may result from the instability of the β-diketone group in the curcumin structure. Due to the electron-withdrawing effect of two carbonyl groups, the acidity of hydrogen atom on methylene is enhanced. Meanwhile, the highly conjugated structure of curcumin, the negative charge after proton removal is highly dispersed, so the stability of curcumin in alkaline environment is poor [10].

A series of curcumin analogs modification on β-diketone moeity were synthesized that included: (1) curcumin analogs that retained the 7-carbon spacer between the aryl rings; (2) curcumin analogs with a 5-carbon spacer, and (3) curcumin analogs with a 3-carbon spacer (chalcones) [11,12]. The data showed that analogs with a 5-carbon spacer can markedly increase the biological activity of curcumin analogs [13]. Therefore, mono-carbonyl analogs of curcumin show better pharmacokinetic properties and bioactivities than curcumin and may represent novel therapeutic entities for the treatment of tumor diseases [14], while substituents on the hydroxy on benzene of curcumin may represent an important pharmacophore for biological activity [15]. The curcumin analog dimethylcurcumin (ASC-J9), which has a methoxy group at the hydroxy on benzene had enhanced cytotoxicity against prostate cancer cell lines [16]. The structure–activity relationship (SAR) analysis of curcumin indicated that modifications mainly focused on the β-diketone structure and hydroxy on benzene could largely enhance stability, bioavailability, and antitumor activity [17,18].

In this study, we attempt to design new curcumin analogs modified on the β-diketone structure and hydroxy on benzene to increase the anti-human hepatocellular carcinoma (HCC) activity. On the one hand, the β-diketone structure was changed to a single ketone or chalcone structure to enhance molecular stability. On the other hand, natural products such as cinnamic acid analogs with strongly lipid solubility were introduced to the hydroxy on benzene to improve solubility and bioavailability [19]. Through the synergistic effect of curcumin and cinnamic acid, the compounds with favorable bioavailability and potent anti-HCC activity could be obtained. This strategy may provide some reference values for the research and development of curcumin analogs with clinical potential.

## 2. Results and Discussion

### 2.1. Chemistry

**Scheme 1:**
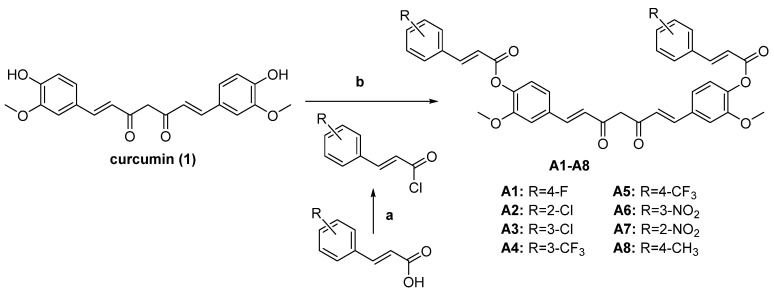
Synthesis of the curcumin analogs **A1**–**A8**. Reagents and conditions: (**a**) SOCl_2_, 80 °C, reflux, 2 h; (**b**) CHCl_3_, triethylamine, 0 °C to room temperature, 12 h.

**Scheme 2:**
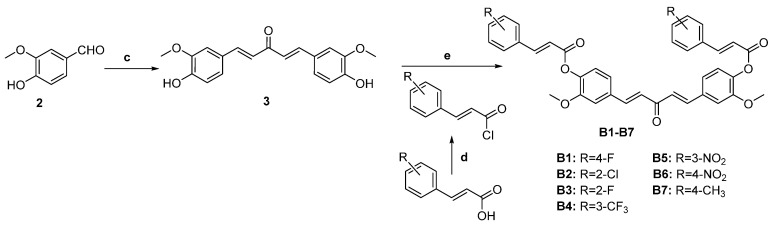
Synthesis of the curcumin analogs **B1**–**B7**. Reagents and conditions: (**c**) acetone, aqueous NaOH, ethanol, 48 h; (**d**) SOCl_2_, 80 °C, reflux, 2 h; (**e**) CHCl_3_, triethylamine, 0 °C to room temperature, 12 h.

**Scheme 3:**
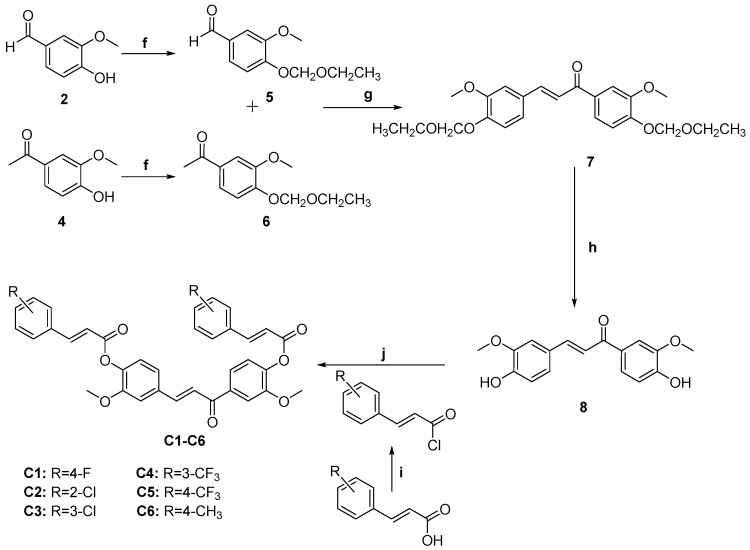
Synthesis of the curcumin analogs **C1**–**C6**. Reagents and conditions: (**f**) chloromethyl ethyl ether, DIEA, CH_2_Cl_2_, 12 h; (**g**) aqueous NaOH, ethanol, 48 h; (**h**) concentrated hydrochloric acid, methanol, 12 h; (**i**) SOCl_2_, 80 °C, reflux, 2 h; (**j**) CHCl_3_, triethylamine, 0 °C to room temperature, 12 h.

To expand the structure diversity, three kinds of curcumin analogs were first synthesized by the procedures in Scheme 1, Scheme 2 and Scheme 3, respectively. As shown in Scheme 1, curcumin (**1**) was reacted with cinnamic acid analogs to give yellow compounds **A1**–**A8** through esterification in CHCl_3_ containing triethylamine at 0 °C with constant stirring for 12 h. As shown in Scheme 2, aqueous NaOH was added dropwise to the solution of vanillic aldehyde (**2**) and acetone in anhydrous ethanol and the pale yellow intermediate (**3**) was produced by an aldol condensation reaction for 48 h. The synthesis of compounds **B1**–**B7** from intermediate **3** was then carried out by the same procedure as described for the preparation of **A1**–**A8**. As shown in Scheme 3, synthesis of the curcumin analogs **C1**–**C6** consists of four main steps, including the protection of hydroxy on benzene of vanillic aldehyde (**2**) or vanilla ketone (**4**) by chloromethyl ethyl ether and N,N-diisopropylethylamine (DIEA), the aldol condensation reaction to synthesize **7** from the corresponding intermediate **5** and **6**, the deprotection by concentrated hydrochloric acid, and then esterification to obtain white compounds.

### 2.2. Novel Curcumin Analogs Strongly Inhibit Proliferation in HepG2 Cell

**Figure 1:**
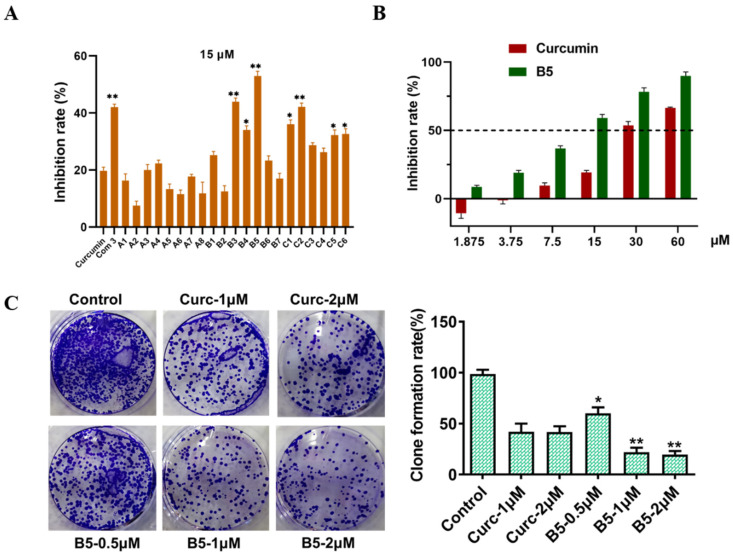
Anti-proliferation assay to HepG2 cells in vitro. (**A**) Curcumin analogs at the concentration of 15 μM for 48 h. (**B**) **B5** at the different concentrations (1.875, 3.75, 7.5, 15, 30, 60 μM) for 48 h. (**C**) Clone formation assay to HepG2 cells treatment with DMEM medium containing the indicated concentrations (0.5, 1, 2 μM) of curcumin and **B5** for 2 weeks. Data are represented by the mean ± SD of the three independent experiments. ** *p* < 0.01, * *p* < 0.05, compared with the control group.

The cytotoxic activity of curcumin analogs towards human hepatocellular carcinoma cell (HepG2) was evaluated by MTT assay [20]. The cell viability was reduced by approximately 20% treated with 15 μM curcumin for 48 h compared with that in the DMEM control group. Curcumin and intermediate 3 were used as positive controls. Thus, inhibition of cell proliferation was determined 48 h after exposure to 15 μM of each compound. The anti-proliferative results are summarized in Figure 1. As shown in Figure 1A, curcumin analogs in group B and group C showed stronger anti-proliferation activities than groups A except **B2** in general, the main reason could account for the stability of single ketone and chalcone. Remarkably, **B5** exhibited the most potent anti-proliferation activity with 52.90 % inhibition against only the DMEM medium added. **B3** (43.98 %) and **C2** (42.19 %) showed moderate potency to curcumin (19.77 %). The analogs with electron-withdrawing groups such as fluorine, trifluoromethyl, and nitro on benzene ring exhibited more effective inhibitory activities than the analogs with electron-donating groups such as methyl comparing **B1** and **B3**–**B5** to **B7** in group B, and the coincident results appeared in group A. Introducing fluorine and nitro in *o*-substitutions showed higher inhibitory activities than chlorine when compared **A7** to **A2** and **B3** to **B2**. Introducing nitro in *m*-substitutions exhibited the strongest inhibitory activities when compared **B5** to **B4**, while the contrary results appeared in group A. The *o*-substitutions and *p*-substitutions showed stronger activity than *m*-substitutions in group C. As shown in Figure 1B, the promising analog **B5** was subsequently selected to investigate the IC_50_ values with different concentrations (1.875, 3.75, 7.5, 15, 30, 60 μM) for 48 h. The IC_50_ values of **B5** and curcumin were 11.33 and 32.83 μM, respectively. As shown in Figure 1C, the clone formation assay [21] also showed that curcumin analog **B5** inhibited the clone formation ability of HepG2 cells. The results of MTT and clone formation assay indicated that **B5** showed promising anti-proliferation activity in HepG2 cells.

### 2.3. Inhibition Effect on HepG2 Cell Migration

**Figure 2:**
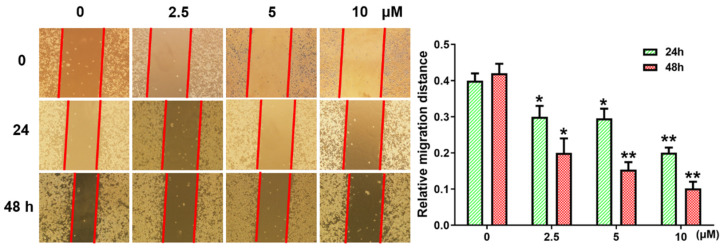
Wound healing assay to HepG2 cells treatment with DMEM medium containing the indicated concentrations (0, 2.5, 5, 10 μM) of **B5** for 24 h and 48 h. Data are represented by the mean ± SD of the three independent experiments. ** *p* < 0.01, * *p* < 0.05, compared with the 0 μM group.

Cancer cell migration plays a critical role in invasion and metastasis, inhibition of cancer cell migration is associated with suppression of cancer progression [22]. Therefore, we investigated the effect of **B5** on HepG2 cell migration. HepG2 cells were treated with **B5** (0, 2.5, 5, 10 μM) and wound healing area/migrated cells in the gap were determined after 24 h and 48 h performing wound/scratch at 0 h. Wound healing assay showed that HepG2 cells treated with **B5** migrated much slower as compared to the control cells in a dose- and time-dependent manner (Figure 2). The result suggested that **B5** can suppress HepG2 cell migration and thereby inhibit liver cancer progression.

### 2.4. Apoptosis of HepG2 Cells through Mitochondrial Death Pathway Generation

**Figure 3:**
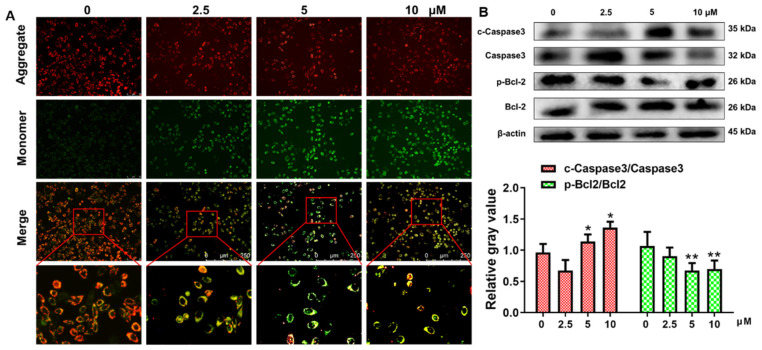
(**A**) The depolarization of mitochondrial transmembrane potential (ΔΨ_m_) could be induced by compound **B5** (0, 2.5, 5, 10 μM) for 48 h, and then stained with JC-1 for 20 min at 37 °C. Both the loss of red fluorescence and the gain of green-emitting monomers indicated the disruption of the mitochondrial transmembrane potential (ΔΨ_m_). The representative images are shown of the corresponding fluorescent channel (40× magnification). (**B**) HepG2 cells were treated with compound **B5** (0, 2.5, 5, 10 μM) for 48 h. Apoptosis-related proteins Bcl-2 and caspase 3 protein levels were determined by Western blotting. Image J software (version 1.8.0, National Institutes of Health, Bethesda, MD, USA) was used to analyze the levels of Bcl-2 and caspase 3 with β-actin as the reference. Data are represented by the mean ± SD of the three independent experiments. ** *p* < 0.01, * *p* < 0.05, compared with the 0 μM group.

Mitochondria play a crucial role in the induction and control of apoptosis through the release of proapoptotic factors and other apoptosis-inducing factors. Dissipation of the mitochondrial electrochemical potential gradient is known as an early event in apoptosis [23]. The depolarization of mitochondrial transmembrane potential (ΔΨ_m_) could be induced by the opening of the mitochondrial permeability transition pore when apoptosis happens. The metachromatic fluorochrome JC-1 has been widely used for the detection of mitochondrial transmembrane potential (ΔΨ_m_) depolarization. JC-1 aggregates in the mitochondrial matrix emitting red fluorescence for intact mitochondria with normal ΔΨ_m_, whereas apoptosis induced the loss of red fluorescence and the gain of green-emitting monomers at depolarized abnormal ΔΨ_m_ [24]. We treated HepG2 cells with compound **B5** (0, 2.5, 5, 10 μM) for 48 h and then stained with JC-1 for 20 min at 37 °C. The JC-1 staining demonstrated that **B5** resulted in red fluorescence loss while green fluorescence accumulation indicated that **B5** dissipated the mitochondrial transmembrane potential (ΔΨ_m_) (Figure 3A).

The Bcl-2 family and caspase family are two important components of the mechanism of apoptosis. The members of the Bcl-2 family both proapoptotic proteins and antiapoptotic such as Bcl-2 protein play a pivotal role in the regulation of the mitochondrial apoptotic pathway [25]. Apoptotic caspases are divided into two categories: initiator caspases and executioner caspases such as caspase 3, which amplifies apoptotic signaling downstream [26]. We exposed HepG2 cells to compound **B5** (0, 2.5, 5, 10 μM) for 48 h and then analyzed the expression of apoptosis-related proteins by Western blotting. It was observed that the ratio of p-Bcl-2/Bcl-2 protein was down-regulated and c-caspase 3/caspase 3 protein was up-regulated as shown in Figure 3B.

These data demonstrated that compound **B5** can induce apoptosis in the liver cell in a dose-dependent manner via depolarizing mitochondrial transmembrane potential (ΔΨ_m_) and the mitochondrial death pathway generation.

### 2.5. Curcumin-Induced Comprehensive Transcriptomes Profile in HepG2 Cells

**Figure 4:**
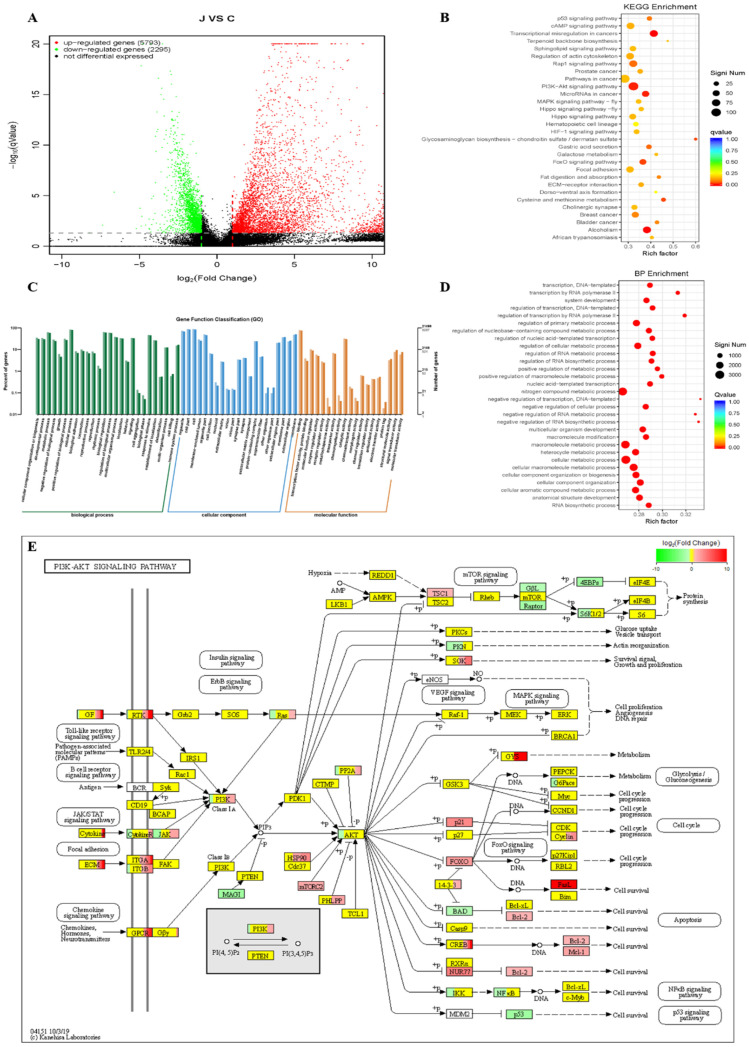
Transcriptome alterations of HepG2 cells were uncovered by high-throughput sequencing. (**A**) Volcano map showed significant differentiation of gene expression in HepG2 cells treated with or without curcumin (*n* = 3). The larger the –log_10_ (pValue), the more significant difference. The red, green, and black spots in the scatter plot showed up-regulated, down-regulated, and no difference genes, respectively. (**B**) The top 30 DEGs were provided in Table 1 via KEGG enrichment analysis. PI3K-Akt signaling pathway (ID ko04151) is significant (109/1805) in cancer. The Qvalue was represented by the color of the spots, and the smaller the Qvalue, the closer to red. The number of DEGs in each function was represented by the size of the spots. (**C**) Histogram of GO annotation classification of differential genes. GO enrichment consists of three ontologies: molecular function (MF), cellular component (CC), and biological process (BP). The light colors represented the differentially expressed genes and the dark colors represented all genes. (**D**) The top 30 DEGs were provided via BP enrichment analysis. (**E**) The AKT signal pathway. The brightness of the color reflected the average expression level of the certain gene via KEGG enrichment analysis. The red indicated relatively higher gene expression while the green represented lower gene expression.

A large number of studies have unraveled that curcumin suppressed cancer development via the regulation of various biological molecules and activation of specific signal pathways. To explore the underlying mechanism of curcumin, we drew a comprehensive transcriptomes profile by high throughput sequencing to identify changes in gene expression in HepG2 cells treated with curcumin. Analysis of sequencing data between the curcumin-untreated or -treated groups indicated that a total of 5793 differentially expressed genes (DEGs) were significantly up-regulated and 2295 genes down-regulated in the curcumin-treated group in Figure 4A. To determine the possible functions of enriched genes and signal pathways correlated with the anti-tumor activities of curcumin, Kyoto Encyclopedia of Genes and Genomes (KEGG) enrichment analysis (Figure 4B,E) and Gene Ontology (GO) enrichment analysis were applied to explore the role of DEGs under curcumin treatment (Figure 4C,D) [27]. KEGG pathway enrichment analysis could find the significant pathways and GO enrichment analysis further assessed the biological process. The result demonstrated that the most enriched terms were relevant to the biological process on the regulation of cellular metabolic process and AKT signaling pathway. These DEGs and related pathways may improve the understanding of the mechanism by which curcumin inhibits HepG2 cell proliferation, migration, and induces apoptosis. We could conclude that curcumin and its analogs have influence on HepG2 cell metabolic process through inhibition of the expression of AKT protein.

**Table 1:** The top 30 DGEs via KEGG enrichment analysis.

ID	Description	Significant	Annotated	Pvalue	Qvalue
ko05202	Transcriptional misregulation in cancers	70/1805	169/7736	1.00 × 10^−7^	3.00 × 10^−5^
ko05034	Alcoholism	69/1805	180/7736	3.67 × 10^−6^	0.000551
ko04151	PI3K-Akt signaling pathway	109/1805	337/7736	7.10 × 10^−5^	0.007097
ko05206	MicroRNAs in cancer	52/1805	138/7736	9.58 × 10^−5^	0.007186
ko00532	Glycosaminoglycan biosynthesis—chondroitin sulfate/dermatan sulfate	12/1805	20/7736	0.000469	0.027069
ko04068	FoxO signaling pathway	46/1805	126/7736	0.000541	0.027069
ko00270	Cysteine and methionine metabolism	21/1805	46/7736	0.000698	0.029904
ko04971	Gastric acid secretion	29/1805	74/7736	0.001596	0.059848
ko04015	Rap1 signaling pathway	67/1805	208/7736	0.001903	0.063443
ko04115	p53 signaling pathway	26/1805	66/7736	0.002502	0.075074
ko05219	Bladder cancer	18/1805	42/7736	0.003896	0.092958
ko05224	Breast cancer	49/1805	148/7736	0.004009	0.092958
ko04975	Fat digestion and absorption	17/1805	39/7736	0.004028	0.092958
ko04512	ECM-receptor interaction	30/1805	84/7736	0.006771	0.140346
ko04810	Regulation of actin cytoskeleton	65/1805	211/7736	0.007017	0.140346
ko04024	cAMP signaling pathway	61/1805	197/7736	0.007824	0.144381
ko05215	Prostate cancer	30/1805	85/7736	0.008182	0.144381
ko04510	Focal adhesion	60/1805	195/7736	0.009586	0.154245
ko04390	Hippo signaling pathway	47/1805	147/7736	0.009769	0.154245
ko05200	Pathways in cancer	111/1805	392/7736	0.01089	0.161349
ko00052	Galactose metabolism	14/1805	33/7736	0.011553	0.161349
ko00900	Terpenoid backbone biosynthesis	10/1805	21/7736	0.012603	0.161349
ko04391	Hippo signaling pathway-fly	25/1805	70/7736	0.01275	0.161349
ko04066	HIF-1 signaling pathway	32/1805	95/7736	0.013676	0.161349
ko04013	MAPK signaling pathway-fly	28/1805	81/7736	0.014076	0.161349
ko05143	African trypanosomiasis	15/1805	37/7736	0.014586	0.161349
ko04071	Sphingolipid signaling pathway	41/1805	128/7736	0.01462	0.161349
ko04725	Cholinergic synapse	36/1805	110/7736	0.015059	0.161349
ko04640	Hematopoietic cell lineage	28/1805	84/7736	0.023258	0.240598
ko04320	Dorso-ventral axis formation	11/1805	26/7736	0.024647	0.245998

### 2.6. The Inhibitory Mechanism of Anti-HCC Activity of Curcumin and B5 toward HepG2 Cells

**Figure 5:**
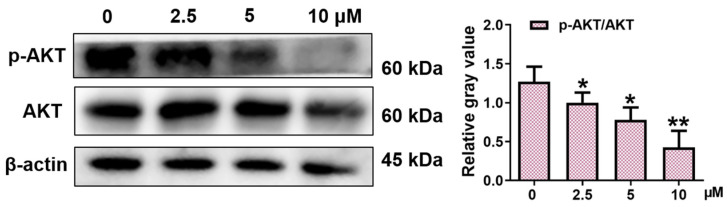
Effect of compound **B5** on Akt protein at the cell level. HepG2 cells were pretreated with compound **B5** (0, 2.5, 5, 10 μM) for 48 h, cell lysates were prepared and analyzed for the phosphorylation state of the Akt kinase via Western blot. Data are represented by the mean ± SD of the three independent experiments. ** *p* < 0.01, * *p* < 0.05, compared with the 0 μM group.

Curcumin-induced comprehensive transcriptomes profile in HepG2 cells indicated that the AKT signal pathway plays a pivotal role in understanding the mechanism of anti-HCC activity of curcumin and its analogs. To further evaluate the effect of the compound **B5** on cellular AKT signaling, the AKT protein in HepG2 cell was examined in the presence or absence of representative compound **B5**, which was found to be the most potent compound against HepG2 cell growth (IC_50_ = 11.33 μM) in current work. After being treated with various concentrations of **B5** (0, 2.5, 5, 10 μM), the HepG2 cell was lysed and analyzed by Western blot [28]. As shown in Figure 5, the phosphorylation of AKT was significantly suppressed by compound **B5** in a dose-dependent manner. This result indicated curcumin analog **B5** can block AKT function at the cell level.

**Figure 6:**
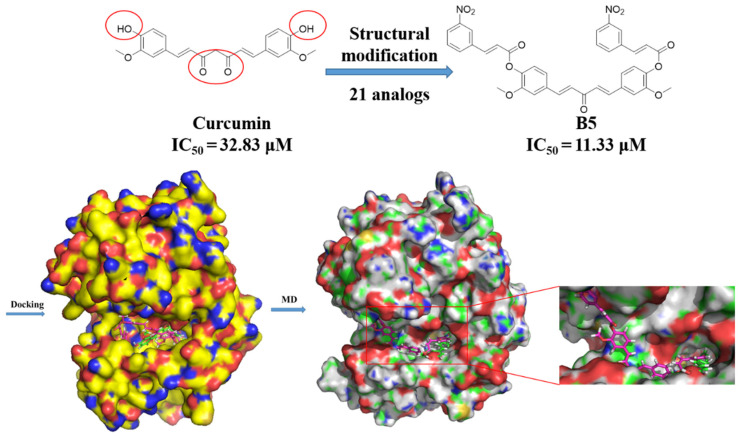
Interactions pattern between small molecules (curcumin and **B5**) and AKT protein showed the electrostatic surface in the 3D representation from molecular docking. Curcumin (green) and **B5** (pink) were represented in sticks and colored by atom type.

To further explore the interactions of these novel curcumin analogs with AKT protein, a molecular docking technique was carried out via AutoDock 4.2 software (Scripps Research, San Diego, CA, USA). The crystal structure (PDB ID: 3MV5, resolution: 2.47 Å) of AKT was downloaded from the Protein Data Bank [29,30]. Curcumin and **B5** were docked to AKT protein (Figure 6). The results showed that curcumin and **B5** adopt a propeller-shaped conformation in the propeller-shaped pocket of the ATP binding site, which is in agreement with previous research [31]. Compound **B5** was well extended into the active pocket and showed the stronger binding affinity (−6.28 Kcal/mol) to AKT protein than curcumin (−3.57 Kcal/mol), which was conducive to playing an important role in protein inhibition and stability.

**Figure 7:**
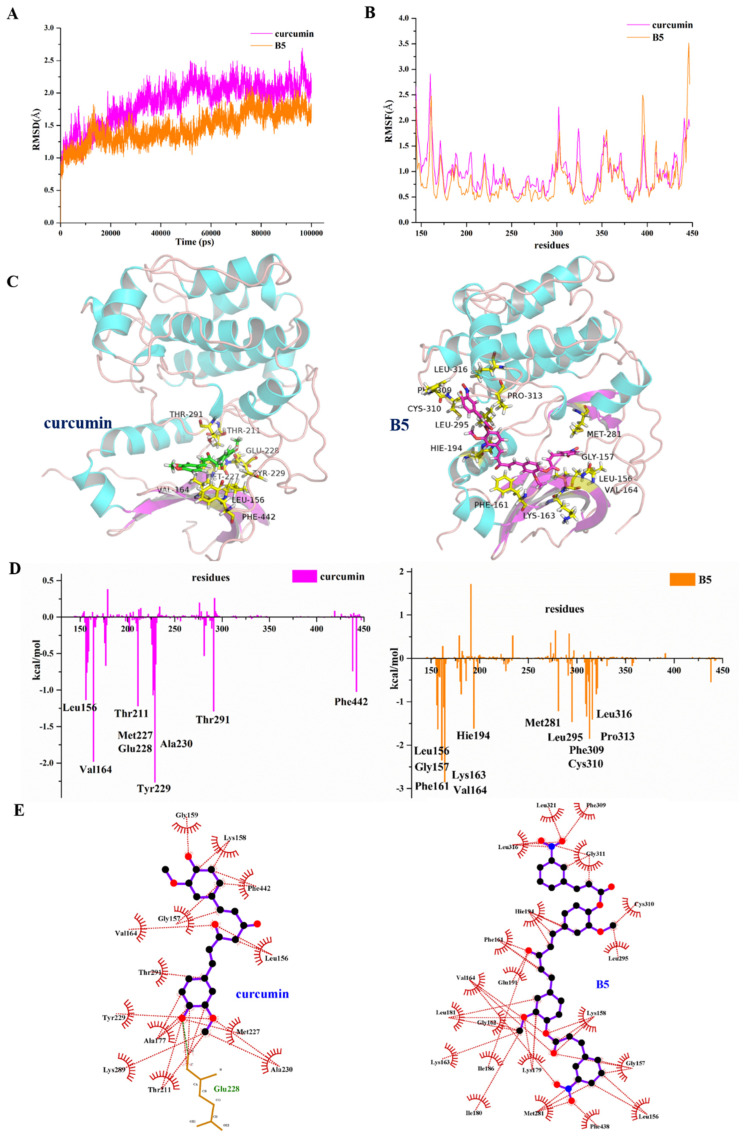
Predicted binding mechanism of AKT in complex curcumin or B5 after 100 ns MD simulations. (**A**) RMSD changes. (**B**) RMSF changes. (**C**) The conformations and specific binding modes. (**D**) Decomposition free energy calculated by MM-GBSA. (**E**) hydrophobic interaction.

To further understand the reasonable binding modes of curcumin and **B5** in complex with AKT, 100 ns molecular dynamics (MD) simulations were performed (Figure 6 and Figure 7). As shown in Figure 7A, the root mean square deviations (RMSD) of AKT-**B5** and AKT-curcumin were ultimately maintained around 1.7 Å and 2.1 Å, respectively, which revealed that equilibrated and converged states were achieved after 100 ns simulation. These results suggested that the complexes underwent reasonable conformational changes. The atomic fluctuation of protein residues was measured by root mean square fluctuation (RMSF) and the results showed that the active site residues showed stable and low fluctuation (Figure 7B). The conformations and specific binding modes of curcumin and **B5** with AKT at 100 ns of MD simulations are shown in Figure 7C.

To further explore the cause of the inhibitory potency against AKT, we performed binding free energy analyses of curcumin and **B5**. The equilibrium stage from the last 2 ns MD trajectories was used for the MM/GBSA calculation. As shown in Table 2, the binding free energy (ΔG_total_) results were in agreement with the experimental values of curcumin and **B5**, which the value of **B5** (ΔG_total_ = −55.02 ± 3.22 kcal/mol) was more than twice as much as curcumin (ΔG_total_ = −27.06 ± 3.22 kcal/mol). The van der Waals interactions (E_vdw_ = −38.18 ± 3.20 and −73.18 ± 3.97 kcal/mol) played a more important role than the electrostatic interaction (E_el_ = −25.84 ± 3.43 and −33.57 ± 7.04 kcal/mol). In addition, the polar interactions (E_el_+E_gb_) of these two systems were greater than the nonpolar interactions (E_vdw_+E_surf_), suggesting that the nonpolar interactions in the two systems, especially E_vdw_, were more favorable for the binding of curcumin and **B5** to AKT, which is in agreement with the stronger hydrophobic interaction.

To investigate the detailed contributions of residues in AKT-curcumin and AKT-**B5** systems, decomposition free energy calculations were carried out by using MM/GBSA methods. As shown in Figure 7D, curcumin has hydrogen bonds formed with hinge region residues Glu 228 (−0.98 kcal/mol) and Ala 230 (−0.65 kcal/mol), which is in agreement with previous research [28]. Although **B5** cannot form hydrogen bonds with residues Glu 228 and Ala 230 like curcumin, compound **B5** formed the stronger interaction with key residues than curcumin in the active pocket and the number of key residues (ΔG_total_ < −1.0 Kcal/mol) of **B5** was more than curcumin. The binding energy and key residues (<−1.0 Kcal/mol) were illustrated in Table 3. Compound **B5** formed the strongest interaction with residue Val164 (−2.85 kcal/mol) and formed interactions with LEU156, GLY157, PHE161, LYS163, VAL164, and MET281 in the deep site of the active pocket. Due to its structure of a longer hydrophobic chain, **B5** formed the strong hydrophobic interaction with HIE194, LEU295, PHE309, CYS310, PRO313, and LEU316 in Figure 7E. The hydrophobic interaction made the structure of **B5** stretch more and further occupied the substrate hydrolysis pathway of the binding cavity. This might be one of the reasons why the inhibitory activity of **B5** was higher than that of curcumin. However, the lack of hydrogen bonds with key residues Glu 228 and Ala 230 explained that the overall potency of **B5** was in the micromolar range and exhibited moderate stronger inhibitory activity than curcumin. This suggested the subsequent structural optimization may focus on shortening the carbon chain modification on hydroxy on benzene suitably. 

## 3. Experiments

### 3.1. General

^1^H NMR and ^13^C NMR spectra were recorded on Bruker AM 600 MHz and 400 MHz spectrometers with tetramethylsilane (TMS) as internal standard. Melting points (MP) were recorded on an SRS OptiMelt-100 fully automatic micro melting point instrument. Electrospray ionization mass spectra (ESI-MS) were recorded on an Agilent 6520B Q-TOF system. Column chromatography (CC) was performed on silica gel (200–300 mesh; Qingdao Makall Group Co., Ltd.; Qingdao, China). Reactions were monitored by thin-layer chromatography (TLC) on a glass plate coated with silica gel with the fluorescent indicator (GF254). Reagents were analytical reagent grade and purchased from Aansoole.

### 3.2. General Procedure for the Preparation of Curcumin Analogs **A1**–**A8**

The starting materials (cinnamic acid analogs) for the synthesis of esters should be activated in the first procedure: the cinnamic acid analogs (1 mmol) and SOCl_2_ (5 mL) were mixed and stirred at reflux 80 °C for 2 h. The reaction mixture was cooled and evaporated to give reactive acyl chloride obtained as an oil, which would be dissolved in CHCl_3_ in the next step. A solution of acyl chloride in CHCl_3_ was added dropwise to curcumin (compound **1**, 0.3 mmol) in CHCl_3_ containing triethylamine at 0 °C with constant stirring for 12 h. The reaction mixture was then neutralized by adding 1 N HCl and extracted with CH_2_Cl_2_. The combined organic layer was dried over anhydrous sodium sulphate and was purified by a flash chromatography with methanol/CH_2_Cl_2_ to give these yellow compounds **A1**–**A8** (Figure S1). The yields were between 55% and 65%.

#### 3.2.1. ((1E,6E)-3,5-dioxohepta-1,6-diene-1,7-diyl)bis(2-methoxy-4,1-phenylene) (2E,2′E)-bis(3-(4-fluorophenyl)acrylate) (**A1**)

ESI-MS *m*/*z*: 663.66 (M-H)^−^. M.P.: 184–186 °C. ^1^H NMR (600 MHz, DMSO-*d*_6_) δ 7.92–7.88 (m, 4H), 7.86 (s, 2H), 7.68 (d, *J* = 15.9 Hz, 2H), 7.55 (s, 2H), 7.36 (d, *J* = 8.2 Hz, 2H), 7.30 (t, *J* = 8.8 Hz, 4H), 7.24 (d, *J* = 8.1 Hz, 2H), 7.02 (d, *J* = 15.9 Hz, 2H), 6.87 (d, *J* = 16.1 Hz, 2H), 6.22 (s, 1H), 3.85 (s, 6H). ^13^C NMR (151 MHz, DMSO-*d*_6_) δ 183.23, 164.19, 162.78, 151.32, 145.54, 140.92, 139.87, 133.78, 131.15, 130.52, 124.65, 123.44, 121.50, 116.59, 116.13, 115.98, 112.09, 101.78, 56.00.

#### 3.2.2. ((1E,6E)-3,5-dioxohepta-1,6-diene-1,7-diyl)bis(2-methoxy-4,1-phenylene) (2E,2′E)-bis(3-(2-chlorophenyl)acrylate) (**A2**)

ESI-MS *m*/*z*: 696.13 (M-H)^−^. M.P.: 167–170 °C. ^1^H NMR (400 MHz, DMSO-*d*_6_) δ 8.14–8.07 (m, 2H), 7.95–7.85 (m, 4H), 7.60 (d, *J* = 8.8 Hz, 2H), 7.54 (d, *J* = 7.5 Hz, 2H), 7.47–7.39 (m, 4H), 7.36 (d, *J* = 11.7 Hz, 2H), 7.31–7.14 (m, 2H), 7.00 (dd, *J* = 15.9, 9.9 Hz, 2H), 6.61 (d, *J* = 15.9 Hz, 2H), 6.15 (s, 1H), 3.87 (s, 6H). ^13^C NMR (151 MHz, DMSO-*d*_6_) δ 183.20, 164.17, 162.88, 151.33, 145.55, 140.90, 139.86, 133.80, 131.17, 130.54, 124.66, 123.46, 121.52, 116.57, 116.11, 115.99, 112.11, 101.79, 56.01.

#### 3.2.3. ((1E,6E)-3,5-dioxohepta-1,6-diene-1,7-diyl)bis(2-methoxy-4,1-phenylene) (2E,2′E)-bis(3-(3-chlorophenyl)acrylate) (**A3**)

ESI-MS *m*/*z*: 696.56 (M-H)^−^. M.P.: 174–177 °C. ^1^H NMR (400 MHz, DMSO-*d*_6_) δ 7.96 (s, 2H), 7.86 (d, *J* = 16.0 Hz, 2H), 7.80 (d, *J* = 7.3 Hz, 2H), 7.68 (d, *J* = 15.9 Hz, 2H), 7.51 (td, *J* = 16.4, 15.4, 8.6 Hz, 6H), 7.37 (d, *J* = 8.2 Hz, 2H), 7.26 (d, *J* = 8.1 Hz, 2H), 7.02 (dd, *J* = 16.0, 7.0 Hz, 4H), 6.22 (s, 1H), 3.86 (s, 6H). ^13^C NMR (101 MHz, DMSO-*d*_6_) δ 183.22, 163.99, 151.26, 145.12, 140.82, 139.85, 136.05, 133.82, 130.76, 130.51, 128.29, 127.30, 124.68, 123.40, 121.50, 118.45, 112.10, 101.77, 56.00.

#### 3.2.4. ((1E,6E)-3,5-dioxohepta-1,6-diene-1,7-diyl)bis(2-methoxy-4,1-phenylene) (2E,2′E)-bis(3-(3-(trifluoromethyl)phenyl)acrylate) (**A4**)

ESI-MS *m*/*z*: 763.67 (M-H)^−^. M.P.: 177–179 °C. ^1^H NMR (400 MHz, DMSO-*d*_6_) δ 8.24 (s, 2H), 8.16 (d, *J* = 7.7 Hz, 2H), 7.98 (d, *J* = 16.1 Hz, 2H), 7.82 (d, *J* = 7.8 Hz, 2H), 7.74–7.65 (m, 4H), 7.57 (s, 2H), 7.38 (d, *J* = 8.2 Hz, 2H), 7.27 (d, *J* = 8.2 Hz, 2H), 7.11 (d, *J* = 16.1 Hz, 2H), 7.03 (d, *J* = 16.0 Hz, 2H), 6.23 (s, 1H), 3.86 (s, 6H). ^13^C NMR (101 MHz, DMSO-*d*_6_) δ 183.23, 163.97, 151.26, 144.99, 140.82, 139.85, 135.00, 133.85, 132.25, 130.04, 129.71, 125.52, 124.70, 123.39, 122.61, 121.52, 118.96, 112.12, 101.77, 56.02.

#### 3.2.5. ((1E,6E)-3,5-dioxohepta-1,6-diene-1,7-diyl)bis(2-methoxy-4,1-phenylene) (2E,2′E)-bis(3-(4-(trifluoromethyl)phenyl)acrylate) (**A5**)

ESI-MS *m*/*z*: 763.18 (M-H)^−^. M.P.: 219–221 °C. ^1^H NMR (400 MHz, DMSO-*d*_6_) δ 8.06 (d, *J* = 8.0 Hz, 4H), 7.95 (d, *J* = 16.1 Hz, 2H), 7.82 (d, *J* = 8.1 Hz, 4H), 7.69 (d, *J* = 15.9 Hz, 2H), 7.57 (s, 2H), 7.38 (d, *J* = 8.1 Hz, 2H), 7.27 (d, *J* = 8.2 Hz, 2H), 7.06 (t, *J* = 16.5 Hz, 4H), 6.23 (s, 1H), 3.86 (s, 6H). ^13^C NMR (101 MHz, DMSO-*d*_6_) δ 183.21, 163.99, 151.24, 145.01, 140.80, 139.83, 135.01, 133.84, 132.23, 130.02, 129.70, 125.51, 124.71, 123.40, 122.63, 121.50, 118.94, 112.14, 101.78, 56.02.

#### 3.2.6. ((1E,6E)-3,5-dioxohepta-1,6-diene-1,7-diyl)bis(2-methoxy-4,1-phenylene) (2E,2′E)-bis(3-(3-nitrophenyl)acrylate) (**A6**)

ESI-MS *m*/*z*: 717.18 (M-H)^−^. M.P.: 222–224 °C. ^1^H NMR (400 MHz, DMSO-*d*_6_) δ 8.67 (s, 2H), 8.30 (t, *J* = 8.4 Hz, 4H), 8.03 (d, *J* = 16.1 Hz, 2H), 7.76 (t, *J* = 8.0 Hz, 2H), 7.69 (d, *J* = 15.9 Hz, 2H), 7.57 (s, 2H), 7.38 (d, *J* = 8.3 Hz, 2H), 7.27 (d, *J* = 8.0 Hz, 2H), 7.15 (d, *J* = 16.0 Hz, 2H), 7.03 (d, *J* = 16.0 Hz, 2H), 6.23 (s, 1H), 3.87 (s, 6H). ^13^C NMR (101 MHz, DMSO-*d*_6_) δ 183.22, 163.84, 151.24, 148.38, 144.35, 140.78, 139.84, 135.68, 134.41, 133.88, 130.45, 125.05, 124.71, 123.46, 121.51, 119.75, 112.13, 101.78, 56.02.

#### 3.2.7. ((1E,6E)-3,5-dioxohepta-1,6-diene-1,7-diyl)bis(2-methoxy-4,1-phenylene) (2E,2′E)-bis(3-(2-nitrophenyl)acrylate) (**A7**)

ESI-MS *m*/*z*: 717.67 (M-H)^−^. M.P.: 215–217 °C. ^1^H NMR (400 MHz, DMSO-*d*_6_) δ 8.19–8.02 (m, 6H), 7.83 (t, *J* = 7.5 Hz, 2H), 7.78–7.62 (m, 4H), 7.57 (s, 2H), 7.38 (d, *J* = 7.9 Hz, 2H), 7.28 (d, *J* = 8.0 Hz, 2H), 7.04 (d, *J* = 15.9 Hz, 2H), 6.92 (d, *J* = 15.8 Hz, 2H), 6.23 (s, 1H), 3.87 (s, 6H). ^13^C NMR (101 MHz, DMSO-*d*_6_) δ 183.24, 163.59, 151.23, 148.40, 141.91, 140.67, 139.84, 134.06, 131.44, 129.56, 128.98, 124.86, 123.40, 121.54, 121.11, 112.17, 101.84, 56.08.

#### 3.2.8. ((1E,6E)-3,5-dioxohepta-1,6-diene-1,7-diyl)bis(2-methoxy-4,1-phenylene) (2E,2′E)-bis(3-(p-tolyl)acrylate) (**A8**)

ESI-MS *m*/*z*: 655.73 (M-H)^−^. M.P.: 209–212 °C. ^1^H NMR (400 MHz, DMSO-*d*_6_) δ 7.93 (d, *J* = 7.8 Hz, 2H), 7.83 (d, *J* = 16.0 Hz, 2H), 7.71 (d, *J* = 7.5 Hz, 4H), 7.57 (d, *J* = 13.6 Hz, 2H), 7.40–7.33 (m, 4H), 7.30–7.25 (m, 4H), 7.04 (dd, *J* = 16.0, 7.8 Hz, 2H), 6.83 (d, *J* = 15.9 Hz, 2H), 6.23 (s, 1H), 3.87 (d, *J* = 9.1 Hz, 6H), 2.37 (d, *J* = 9.7 Hz, 6H). ^13^C NMR (101 MHz, DMSO-*d*_6_) δ 183.21, 163.94, 151.23, 144.96, 140.80, 139.83, 135.03, 133.84, 132.24, 130.06, 129.70, 125.53, 124.71, 123.40, 122.60, 121.53, 118.97, 112.10, 101.75, 56.04.

### 3.3. General Procedure for the Preparation of Curcumin Analogs **B1**–**B7**

Aqueous NaOH (2 M) was added dropwise to a vigorously stirred solution of the vanillic aldehyde (compound **2**, 1.52 g, 10 mmol) and acetone (5 mmol) in anhydrous ethanol (10 mL). The reaction solution turned yellow and was subsequently stirred at room temperature for 48 h. The mixture was treated with water and neutralized by the addition of 2 M HCl solution. The aqueous mixture was then extracted with ethyl acetate and the organic phase was washed with aqueous saturated NH_4_Cl solution and brine. The organic layer was separated and dried over anhydrous MgSO_4_, filtered, and concentrated under reduced pressure to give the crude product, which was purified by flash silica chromatography with ethyl acetate/petroleum ether to produce pale yellow intermediate compound **3** (yield 45%).

#### 3.3.1. (1E,4E)-1,5-bis(4-hydroxy-3-methoxyphenyl)penta-1,4-dien-3-one (**3**)

ESI-MS *m*/*z*: 325.35 (M-H)^−^. M.P.: 128–130 °C. ^1^H NMR (400 MHz, DMSO-*d*_6_) δ 9.62 (s, 1H), 7.52 (d, *J* = 16.3 Hz, 1H), 7.30 (s, 1H), 7.13 (d, *J* = 8.2 Hz, 1H), 6.81 (d, *J* = 8.1 Hz, 1H), 6.67 (d, *J* = 16.2 Hz, 1H), 3.82 (s, 3H). ^13^C NMR (101 MHz, DMSO-*d*_6_) δ 197.80, 149.39, 147.95, 143.93, 125.85, 124.33, 123.24, 115.62, 111.26, 55.66.

The synthesis of white compounds **B1**–**B7** (Figure S1). from intermediate **3** was then carried out by the same procedure as described for the preparation of **A1**–**A8**. The yields were between 50% and 60%.

#### 3.3.2. ((1E,4E)-3-oxopenta-1,4-diene-1,5-diyl)bis(2-methoxy-4,1-phenylene) (2E,2′E)-bis(3-(4-fluorophenyl)acrylate) (**B1**)

ESI-MS *m*/*z*: 621.62 (M-H)^−^. M.P.: 145–148 °C. ^1^H NMR (400 MHz, DMSO-*d*_6_) δ 7.93–7.84 (m, 6H), 7.63 (d, *J* = 16.4 Hz, 2H), 7.53 (s, 2H), 7.31 (q, *J* = 8.4 Hz, 6H), 7.23 (d, *J* = 8.1 Hz, 2H), 6.90 (d, *J* = 5.0 Hz, 2H), 6.85 (d, *J* = 4.7 Hz, 2H), 3.84 (s, 6H). ^13^C NMR (101 MHz, DMSO-*d*_6_) δ 198.13, 164.83, 164.15, 162.35, 151.28, 145.51, 142.63, 140.96, 133.52, 131.18, 131.10, 130.50, 127.55, 123.37, 121.67, 116.58, 116.15, 115.93, 111.95, 55.98.

#### 3.3.3. ((E,4E)-3-oxopenta-1,4-diene-1,5-diyl)bis(2-methoxy-4,1-phenylene) (2E,2′E)-bis(3-(2-chlorophenyl)acrylate) (**B2**)

ESI-MS *m*/*z*: 654.12 (M-H)^−^. M.P.: 153–156 °C. ^1^H NMR (400 MHz, DMSO-*d*_6_) δ 7.95–7.85 (m, 4H), 7.64 (d, *J* = 16.3 Hz, 2H), 7.59–7.51 (m, 6H), 7.47–7.39 (m, 6H), 7.25 (s, 2H), 6.89 (d, *J* = 16.4 Hz, 2H), 3.85 (s, 6H). ^13^C NMR (101 MHz, DMSO-*d*_6_) δ 198.14, 167.16, 163.83, 151.20, 142.60, 141.21, 140.80, 138.68, 134.02, 133.67, 133.56, 132.42, 131.85, 131.68, 131.32, 130.10, 129.95, 128.61, 128.24, 127.89, 127.77, 127.63, 123.34, 122.32, 121.67, 119.77, 112.00, 56.04.

#### 3.3.4. ((1E,4E)-3-oxopenta-1,4-diene-1,5-diyl)bis(2-methoxy-4,1-phenylene) (2E,2′E)-bis(3-(2-fluorophenyl)acrylate) (**B3**)

ESI-MS *m*/*z*: 621.18 (M-H)^−^. M.P.: 117–120 °C. ^1^H NMR (400 MHz, DMSO-*d*_6_) δ 7.97 (t, *J* = 7.6 Hz, 2H), 7.89 (d, *J* = 16.2 Hz, 2H), 7.63 (d, *J* = 16.3 Hz, 2H), 7.53 (s, 4H), 7.32 (p, *J* = 7.9, 7.0 Hz, 6H), 7.25 (s, 2H), 6.91 (dd, *J* = 24.0, 16.3 Hz, 4H), 3.84 (s, 6H). ^13^C NMR (101 MHz, DMSO-*d*_6_) δ 198.11, 164.82, 164.13, 162.37, 151.30, 145.50, 142.64, 140.98, 133.51, 131.16, 131.11, 130.51, 127.56, 123.36, 121.68, 116.59, 116.18, 115.94, 111.93, 55.99.

#### 3.3.5. ((1E,4E)-3-oxopenta-1,4-diene-1,5-diyl)bis(2-methoxy-4,1-phenylene) (2E,2′E)-bis(3-(3-(trifluoromethyl)phenyl)acrylate) (**B4**)

ESI-MS *m*/*z*: 721.64 (M-H)^−^. M.P.: 110–112 °C. ^1^H NMR (400 MHz, DMSO-*d*_6_) δ 8.03 (d, *J* = 8.1 Hz, 4H), 7.94 (d, *J* = 16.1 Hz, 2H), 7.80 (d, *J* = 8.3 Hz, 4H), 7.63 (d, *J* = 16.3 Hz, 2H), 7.53 (d, *J* = 1.7 Hz, 2H), 7.33 (dd, *J* = 8.2, 1.7 Hz, 2H), 7.24 (d, *J* = 8.1 Hz, 2H), 7.05 (d, *J* = 16.1 Hz, 2H), 6.88 (d, *J* = 16.4 Hz, 2H), 3.84 (s, 6H). ^13^C NMR (101 MHz, DMSO-*d*_6_) δ 198.14, 163.90, 162.00, 159.53, 151.21, 142.60, 140.83, 138.40, 133.63, 129.62, 127.61, 125.17, 123.35, 121.67, 116.28, 116.07, 111.97, 56.00.

#### 3.3.6. ((1E,4E)-3-oxopenta-1,4-diene-1,5-diyl)bis(2-methoxy-4,1-phenylene) (2E,2′E)-bis(3-(3-nitrophenyl)acrylate) (**B5**)

ESI-MS *m*/*z*: 675.17 (M-H)^−^. M.P.: 154–156 °C. ^1^H NMR (400 MHz, DMSO-*d*_6_) δ 7.82 (d, *J* = 16.0 Hz, 2H), 7.70 (d, *J* = 8.1 Hz, 4H), 7.63 (d, *J* = 16.4 Hz, 2H), 7.52 (d, *J* = 1.8 Hz, 2H), 7.33 (dd, *J* = 8.2, 1.8 Hz, 2H), 7.27 (d, *J* = 8.0 Hz, 4H), 7.22 (d, *J* = 8.1 Hz, 2H), 6.88 (d, *J* = 16.4 Hz, 2H), 6.82 (d, *J* = 16.0 Hz, 2H), 3.83 (s, 6H). ^13^C NMR (101 MHz, DMSO-*d*_6_) δ 198.15, 163.93, 162.02, 159.51, 151.23, 142.61, 140.85, 138.41, 133.64, 129.64, 127.60, 125.16, 123.34, 121.66, 116.30, 116.08, 111.99, 56.02.

#### 3.3.7. ((1E,4E)-3-oxopenta-1,4-diene-1,5-diyl)bis(2-methoxy-4,1-phenylene) (2E,2′E)-bis(3-(p-tolyl)acrylate) (**B6**)

ESI-MS *m*/*z*: 675.63 (M-H)^−^. M.P.: 152–155 °C. ^1^H NMR (400 MHz, DMSO-*d*_6_) δ 8.28 (d, *J* = 8.8 Hz, 4H), 8.11 (d, *J* = 8.8 Hz, 4H), 7.98 (d, *J* = 16.1 Hz, 2H), 7.64 (d, *J* = 16.4 Hz, 2H), 7.54 (d, *J* = 1.7 Hz, 2H), 7.35 (dd, *J* = 8.2, 1.7 Hz, 2H), 7.26 (d, *J* = 8.1 Hz, 2H), 7.13 (d, *J* = 16.1 Hz, 2H), 6.89 (d, *J* = 16.4 Hz, 2H), 3.84 (s, 6H). ^13^C NMR (101 MHz, DMSO-*d*_6_) δ 198.16, 163.92, 162.03, 159.50, 151.22, 142.60, 140.83, 138.43, 133.66, 129.62, 127.59, 125.14, 123.33, 121.67, 116.31, 116.06, 112.00, 56.02.

#### 3.3.8. ((1E,4E)-3-oxopenta-1,4-diene-1,5-diyl)bis(2-methoxy-4,1-phenylene) (2E,2′E)-bis(3-(p-tolyl)acrylate) (**B7**)

ESI-MS *m*/*z*: 613.69 (M-H)^−^. M.P.: 147–149 °C. ^1^H NMR (400 MHz, DMSO-*d*_6_) δ 7.95 (s, 2H), 7.85 (d, *J* = 16.1 Hz, 2H), 7.79 (d, *J* = 7.5 Hz, 2H), 7.63 (d, *J* = 16.3 Hz, 2H), 7.54–7.46 (m, 6H), 7.35 (d, *J* = 1.7 Hz, 2H), 7.24 (d, *J* = 8.1 Hz, 2H), 7.00 (d, *J* = 16.1 Hz, 2H), 6.88 (d, *J* = 16.4 Hz, 2H), 3.84 (s, 6H). ^13^C NMR (101 MHz, DMSO-*d*_6_) δ 198.11, 163.92, 162.01, 159.54, 151.23, 142.62, 140.85, 138.42, 133.61, 129.61, 127.62, 125.15, 123.32, 121.65, 116.30, 116.05, 111.99, 56.03.

### 3.4. General Procedure for the Preparation of Curcumin Analogs **C1**–**C6**

The hydroxy on benzene should be protected from ether firstly. To a stirred solution of vanillic aldehyde (compound **2**, 1.52 g, 10 mmol) or vanilla ketone (compound **4**, 1.66 g, 10 mmol) in CH_2_Cl_2_ (20 mL) at room temperature was successively added DIEA (5.15 mL, 30 mmol). Then, chloromethyl ethyl ether (1.9 mL, 20 mmol) in CH_2_Cl_2_ was dropwise added to the mixture over an around 10 min period. The solution was stirred 12 h at room temperature and a saturated aqueous NH_4_Cl (100 mL) was added. The combined organic phase was washed with water and brine. The organic layer was separated and dried over anhydrous MgSO_4_, filtered, and concentrated under reduced pressure to give the corresponding intermediate **5** and **6**, which was not purified to the next step. Compound **7** was synthesized by the same procedure as described for the preparation of compound **3**. To a stirred solution of compound **7** in methanol (10 mL) were added concentrated hydrochloric acid (0.1 mL) at room temperature for 12 h. The mixture was added a saturated aqueous NaHCO_3_ and ethyl acetate. The combined organic phase was washed with water and brine. The organic layer was separated and dried over anhydrous MgSO_4_, filtered, and concentrated under reduced pressure. The crude product **8** was purified by a flash chromatography with methanol/CH_2_Cl_2_ (V/V = 1:50). The yield was 65%.

#### 3.4.1. (E)-1,3-bis(4-hydroxy-3-methoxyphenyl)prop-2-en-1-one (**8**)

ESI-MS *m*/*z*: 299.31 (M-H)^−^. ^1^H NMR (400 MHz, DMSO-*d*_6_) δ 9.99 (s, 1H), 9.62 (s, 1H), 7.83–7.71 (m, 2H), 7.63 (d, *J* = 15.8 Hz, 2H), 7.48 (s, 1H), 7.27 (d, *J* = 8.2 Hz, 1H), 6.91 (d, *J* = 8.2 Hz, 1H), 6.83 (d, *J* = 8.1 Hz, 1H), 3.87 (s, 6H). ^13^C NMR (101 MHz, DMSO-*d*_6_) δ 187.03, 151.67, 149.42, 147.97, 147.79, 143.60, 129.86, 126.52, 123.71, 123.56, 118.72, 115.62, 114.92, 111.80, 111.60, 55.86, 55.71.

The white curcumin analogs **C1**–**C6** (Figure S1). were synthesized by the same procedure as described for the preparation of compound **A1**–**A8**. The yields were between 50% and 60%.

#### 3.4.2. ((E)-3-oxoprop-1-ene-1,3-diyl)bis(2-methoxy-4,1-phenylene) (2E,2′E)-bis(3-(4-fluorophenyl)acrylate) (**C1**)

ESI-MS *m*/*z*: 595.58 (M-H)^−^. M.P.: 202–204 °C. ^1^H NMR (400 MHz, DMSO-*d*_6_) δ 7.95–7.86 (m, 7H), 7.84–7.69 (m, 4H), 7.60–7.51 (m, 1H), 7.41 (d, *J* = 8.2 Hz, 1H), 7.29 (q, *J* = 8.3 Hz, 5H), 6.89 (dd, *J* = 16.0, 8.3 Hz, 2H), 3.90 (d, *J* = 8.2 Hz, 6H). ^13^C NMR (101 MHz, DMSO-*d*_6_) δ 188.14, 167.54, 164.87, 164.39, 164.18, 163.99, 162.38, 161.92, 151.33, 145.78, 145.55, 143.70, 143.36, 142.72, 141.22, 136.54, 133.75, 131.17, 130.59, 123.32, 122.38, 119.13, 116.60, 116.43, 116.17, 115.97, 115.77, 112.76, 112.13, 56.10.

#### 3.4.3. ((E)-3-oxoprop-1-ene-1,3-diyl)bis(2-methoxy-4,1-phenylene) (2E,2′E)-bis(3-(2-chlorophenyl)acrylate) (**C2**)

ESI-MS *m*/*z*: 628.49 (M-H)^−^. M.P.: 170–172 °C. ^1^H NMR (400 MHz, DMSO-*d*_6_) δ 8.11 (dt, *J* = 16.2, 8.1 Hz, 4H), 8.02–7.92 (m, 2H), 7.83–7.72 (m, 3H), 7.62–7.42 (m, 8H), 7.31 (d, *J* = 8.1 Hz, 1H), 7.02 (dd, *J* = 15.9, 8.7 Hz, 2H), 3.91 (d, *J* = 9.0 Hz, 6H). ^13^C NMR (101 MHz, DMSO-*d*_6_) δ 188.13, 163.84, 163.65, 151.24, 143.68, 143.16, 141.45, 141.24, 141.04, 136.64, 134.05, 133.86, 132.45, 131.31, 130.10, 128.62, 127.89, 123.29, 122.40, 119.78, 119.63, 112.82, 112.16, 56.15.

#### 3.4.4. ((E)-3-oxoprop-1-ene-1,3-diyl)bis(2-methoxy-4,1-phenylene) (2E,2′E)-bis(3-(3-chlorophenyl)acrylate) (**C3**)

ESI-MS *m*/*z*: 628.11 (M-H)^−^. M.P.: 155–158 °C. ^1^H NMR (400 MHz, DMSO-*d*_6_) δ 7.96 (d, *J* = 7.3 Hz, 3H), 7.91–7.86 (m, 1H), 7.82–7.77 (m, 5H), 7.57–7.48 (m, 5H), 7.46–7.41 (m, 3H), 7.28 (d, *J* = 8.1 Hz, 1H), 7.02 (dd, *J* = 16.0, 8.6 Hz, 2H), 3.90 (d, *J* = 8.6 Hz, 6H). ^13^C NMR (101 MHz, DMSO-*d*_6_) δ 188.13, 167.35, 163.99, 163.80, 151.28, 145.37, 145.14, 143.69, 143.27, 142.31, 141.14, 136.55, 136.04, 133.82, 133.72, 130.77, 130.65, 129.80, 128.32, 127.81, 127.32, 126.77, 123.30, 122.23, 122.07, 120.98, 118.46, 118.31, 112.78, 112.16, 56.12.

#### 3.4.5. ((E)-3-oxoprop-1-ene-1,3-diyl)bis(2-methoxy-4,1-phenylene) (2E,2′E)-bis(3-(3-(trifluoromethyl)phenyl)acrylate) (**C4**)

ESI-MS *m*/*z*: 695.60 (M-H)^−^. M.P.: 172–174 °C. ^1^H NMR (400 MHz, DMSO-*d*_6_) δ 8.23 (s, 2H), 8.16 (d, *J* = 7.3 Hz, 2H), 7.98 (td, *J* = 14.6, 13.0, 8.3 Hz, 4H), 7.84–7.78 (m, 4H), 7.71 (dd, *J* = 14.6, 6.7 Hz, 3H), 7.56 (d, *J* = 8.2 Hz, 1H), 7.43 (d, *J* = 8.2 Hz, 1H), 7.29 (d, *J* = 8.1 Hz, 1H), 7.13 (dd, *J* = 16.0, 8.9 Hz, 2H), 3.91 (d, *J* = 8.8 Hz, 6H). ^13^C NMR (101 MHz, DMSO-*d*_6_) δ 188.14, 163.97, 163.78, 151.27, 145.23, 145.00, 143.69, 143.26, 141.13, 136.61, 134.99, 133.82, 132.26, 130.05, 129.74, 127.10, 125.63, 125.32, 123.28, 122.62, 122.24, 122.07, 118.96, 118.81, 112.80, 112.17, 56.12.

#### 3.4.6. ((E)-3-oxoprop-1-ene-1,3-diyl)bis(2-methoxy-4,1-phenylene) (2E,2′E)-bis(3-(4-(trifluoromethyl)phenyl)acrylate) (**C5**)

ESI-MS *m*/*z*: 695.16 (M-H)^−^. M.P.: 212–214 °C. ^1^H NMR (400 MHz, DMSO-*d*_6_) δ 8.06 (d, *J* = 5.4 Hz, 4H), 8.03–7.92 (m, 4H), 7.86–7.76 (m, 6H), 7.73 (s, 1H), 7.56 (d, *J* = 8.3 Hz, 1H), 7.44 (d, *J* = 8.2 Hz, 1H), 7.30 (d, *J* = 8.1 Hz, 1H), 7.09 (dd, *J* = 16.1, 8.6 Hz, 2H), 3.91 (d, *J* = 9.2 Hz, 6H). ^13^C NMR (101 MHz, DMSO-*d*_6_) δ 188.14, 163.86, 163.67, 151.26, 145.13, 144.90, 143.69, 143.21, 141.08, 137.81, 136.63, 133.85, 129.36, 125.81, 125.34, 123.28, 122.63, 122.25, 122.08, 119.66, 119.51, 112.80, 112.16, 56.13.

#### 3.4.7. ((E)-3-oxoprop-1-ene-1,3-diyl)bis(2-methoxy-4,1-phenylene) (2E,2′E)-bis(3-(p-tolyl)acrylate) (**C6**)

ESI-MS *m*/*z*: 687.66 (M-H)^−^. M.P.: 173–175 °C. ^1^H NMR (400 MHz, DMSO-*d*_6_) δ 8.02–7.92 (m, 2H), 7.89–7.76 (m, 4H), 7.72 (t, *J* = 5.2 Hz, 4H), 7.59–7.53 (m, 2H), 7.41 (d, *J* = 8.2 Hz, 1H), 7.30–7.26 (m, 4H), 7.22 (d, *J* = 7.7 Hz, 1H), 6.85 (dd, *J* = 16.0, 8.4 Hz, 2H), 3.90 (d, *J* = 8.7 Hz, 6H), 2.36 (s, 6H). ^13^C NMR (101 MHz, DMSO-*d*_6_) δ 188.13, 167.69, 164.32, 164.13, 151.35, 147.02, 146.78, 143.92, 143.70, 143.41, 141.27, 141.16, 141.08, 140.14, 136.49, 133.68, 131.50, 131.11, 129.65, 129.50, 128.75, 128.19, 123.39, 123.30, 122.19, 122.05, 118.09, 115.56, 115.40, 112.74, 112.11, 56.09.

### 3.5. Cell Culture

HepG2 cell line was a gift from Nanjing Forestry University. 3-(4,5-Dimethylthiazol-2-yl)-2,5-diphenyltetrazolium bromide (MTT) were from Sigma. 96-well plates, 24-well plates and 6-well plates (Beyotime Biotechnology) were used for studies. Cells were grown in high glucose Dulbecco′s modified Eagle′s medium (DMEM) containing 10% (*v*/*v*) fetal calf serum and 100 mM penicillin and 100 mM streptomycin. Cells were propagated at 37 °C in a humidified atmosphere containing 5% CO_2_ in air. All novel compounds were dissolved in dimethyl sulfoxide to make stock solutions and kept at −20 °C. The final concentration of the vehicle in the solution never exceeded 0.1% and had no effects on cell viability.

### 3.6. MTT Assay

Cell proliferative assay was carried out using 96-well plate cultures and MTT staining. Briefly, the HepG2 cell line (5000 cells/well) was treated with 15 μM novel curcumin analogs in 96-well culture plates for 48 h. Then, 20 μL of MTT (4 mg/mL MTT in PBS) solution was added to each well, and the microplates were further incubated at 37 °C for 4 h. After carefully removing the medium, the precipitates were dissolved in 200 μL of DMSO in the dark, shaken mechanically for 10 min, and then absorbance values at a wavelength of 540 nm were taken on a Super Microplate Reader (MQX2OO) (BioTek, Winooski, VT, USA). Cell inhibition rate (%) = 1 − [(Experimental group − Blank group)/(Control group − Blank group)] × 100%. After preliminary screening, compound **B5** was then selected to treat with HepG2 cell with different concentrations (1.875, 3.75, 7.5, 15, 30, 60 μM) for 48 h, IC_50_ values were calculated through nonlinear fitting in GraphPad Prism 8 software. The results were determined from replicates of 96 wells from at least three independent experiments.

### 3.7. Clone Formation

HepG2 cells were seeded in a 6-well plate in a density of 1000 cells per well containing 2 mL of 37 °C pre-warmed DMEM culture solution. Plates were incubated at 37 °C and 5% CO_2_ for 12 h. Then, the cells were treated with DMEM medium containing the indicated concentrations (0.5, 1, 2 μM) of curcumin and compound **B5** and then incubated for 2 weeks. Finally, the DMEM culture medium was removed, and the cells were washed with PBS and fixed with 4% paraformaldehyde. Next, 2 mL of 0.1% crystal violet solution was added to each well, and plates were incubated for 20 min. The cells were washed with PBS three times, dried, and photographed, and the clone formation rate was calculated.

### 3.8. Wound Healing Assay

A wound healing test was used to evaluate the effects of novel curcumin analogs on tumor cell motility by HepG2 tumor cell lines. The cells (1.5 × 10^5^ cells/well) were cultured in 24-well plates and grown in DMEM to a nearly confluent cell monolayer. A 10 μL plastic pipette tip was used to draw a linear “wound” in the cell monolayer of each well. The monolayer was then washed twice with serum free DMEM medium to remove debris or detached cells, and test compound **B5** was added at different concentrations (0, 2.5, 5, 10 μM) in serum free DMEM medium and subsequently cultured for 24 h or 48 h depending on the different ability of the cell migration. The wound healing of the scratched cells was photographed under a DMIL LED 3000 inverted microscope (Leica, Wetzlar, Germany) and the effects of curcumin analogs on tumor cell motility were expressed as migration % of 0 h (wound width at exposure time point/wound width at 0 h). The experiments were performed in triplicate.

### 3.9. Mitochondrial Membrane Potential Monitored by JC-1 Staining

HepG2 cells were seeded in 6-well plate and treated with compound **B5** (0, 2.5, 5, 10 μM) for 48 h. They were then washed with PBS, added fresh DMEM medium (1 mL) and JC-1 (5,5′,6,6′-tetrachloro-1,1′,3′,3′-tetraethylbenzimidazolylcarbocyanine iodide, Beyotime, C2006) molecular probe (1 mL). After 20 min incubation at 37 °C, they were rinsed twice with PBS. Visualization of JC-1 aggregates (red fluorescence) and JC-1 monomers (green fluorescence) was done using filter (emission 488 and 550 nm, respectively) by Nikon fluorescence microscope (TE2000-U, Nikon Inc., Tokyo, Japan) at 40× magnification, and an excitation wavelength of 488 nm and an emission wavelength of 550 nm.

### 3.10. Western Blot Analysis

The HepG2 cells on 6-well plates were rinsed twice with cold PBS and lysed in 100 μL RIPA lysis buffer (10 mM HEPES, 2 mM EDTA, 0.1% CHAPS, 5 mM DTT and 1 mM PMSF) on ice for 30 min. Insoluble components of cell lysates were removed by centrifugation at 12,000× *g* for 5 min at 4 °C, and protein concentrations were measured using the BCATM protein quantification kit from Beyotime Biotechnology. Antibodies against AKT (C67E7), Bcl-2 (D55G8), caspase 3 (D3R6Y), phospho-AKT (D9E), phospho-Bcl-2 (5H2), cleaved caspase 3 (5AE1), and β-actin (13E5) were purchased from Cell Signalling Technology. The protein level was determined by Western blotting analysis of 50 μg of cell extract. Briefly, the protein samples were centrifuged (4 °C, 12,000× *g*, 10 min) and boiled for 10 min, then subjected to a 10% SDS-polyacrylamide gel electrophoresis at a constant 20 mA current for 1 h. The resolved proteins were transferred to a PVDF membrane by wet rotation at 70 mV and blocked with a blocking buffer (2% free fat milk, 10 mM Tris-Cl, 50 mM NaCl, 0.1% Tween 20, pH 7.4) at room temperature for 1 h. The membrane was incubated with primary antibodies overnight on the converter at a low temperature (4 °C). The next day, the membrane was washed using the washing buffer (10 mM Tris-Cl, 50 mM NaCl, 0.1% Tween 20, pH 7.4) three times, twice for 5 min and once for 10 min, to remove any nonspecific primary antibody binding. Then, the membrane was incubated with an appropriate dilution of secondary antibody at room temperature for 2 h. After washing three times with washing buffer, the membrane was illuminated with ECL reagent according to the manufacturer’s instructions. A photograph of the gel was taken, and the relative band density was analyzed by optical densitometry using Image J software (version 1.8.0, National Institutes of Health, Bethesda, MD, USA).

### 3.11. Molecular Docking Studies

The molecular docking technique was carried out using the AutoDock 4.2 software (Scripps Research, San Diego, CA, USA) to explore the interaction mode between the curcumin, **B5** and AKT protein. The crystal structure (PDB ID: 3MV5) of AKT was downloaded from the Protein Data Bank and the ligand-free PDB files were prepared with PyMOL software (DeLano Scientific LLC, San Carlos, CA, USA). Curcumin and **B5** were prepared using PRODRG software (GlycoBioChem Ltd, Dundee, UK) to generate and optimize the initial structure [32]. Polar hydrogen atoms and Gasteiger charges were added before generating the PDBQT files of the protein and compounds. A 70 × 70 × 70 Å affinity grid was calculated using AutoGrid4 (Scripps Research, San Diego, CA, USA). All other parameters were set as default and the binding affinity was evaluated by the binding free energy (Kcal/mol). Finally, plausible docking models were selected from the abundant clusters between the ligand and receptor that had lower binding energies.

### 3.12. Molecular Dynamics Simulations

Molecular dynamics (MD) simulations of two systems (curcumin and target compound **B5**) in complex with AKT were performed on the basis of the corresponding docking conformation using the Amber14 package [33]. The ff14SB [34] force field was selected for protein parametrization and GAFF [35] was used for curcumin and **B5**. The complex was immersed in a truncated box with TIP3P [36] water molecules, and Na^+^ was added to reduce the total number of static charges to zero. The conjugate gradient method and the steepest descent method were used to minimize the system energy. Subsequently, the complex was heated gradually from 0 to 300 K in the NVT (constant number of particles, volume, and temperature) [37] ensemble and equilibrated at 300 K. During the sampling process, coordinate trajectories were saved every 2 ps. Finally, a 100 ns MD simulation was performed at a constant temperature of 300 K and a constant pressure of 105 Pa using the PMEMD module [38]. Intermolecular interactions, such as hydrogen bonds and hydrophobic interactions, after the MD simulation were evaluated using PyMOL software (DeLano Scientific LLC, San Carlos, CA, USA).

## 4. Conclusions

To improve the anti-HCC activity and bioavailability, we designed and synthesized novel curcumin analogs, known as **A1**–**A8**, **B1**–**B6**, and **C1**–**C6** modification on the β-diketone structure and introducing cinnamic acid analogs into hydroxy on benzene. **B5** exhibited the more significant activity with (IC_50_ = 11.33 μM) than curcumin (IC_50_ = 32.83 μM) and the structure–activity relationship (SAR) was discussed briefly. A series of pharmacological assays containing cell migration by wound healing assay, cell apoptosis by JC-1 staining, and Annexin V-FITC/PI, the underlying mechanism by comprehensive transcriptomes profile, Western blotting, molecular docking, and molecular dynamics analysis were further explored. The results indicated that curcumin analog **B5** may inhibit cell proliferation and migration and induce apoptosis through AKT signaling pathway and the mitochondrial death pathway. The comprehensive transcriptomes profile, molecular docking, and dynamics analysis further supported that AKT kinase would be one target of curcumin analogs. Compound **B5** is characterized by the presence of nitro group. The nitro group has toxicity issues and is often categorized as a structural alert or a toxicophore, and drugs containing nitro groups have been extensively associated with mutagenicity and genotoxicity. This study produces bases for further deeper in vitro or in vivo studies that could lead to effective anti-HCC agents.

## Figures and Tables

**Table 2:** Binding free energy of AKT with curcumin or B5 calculated by MM-GBSA.

Predicted ΔG (Kcal/mol)							
Compound	E_vdw_	E_el_	E_gb_	E_surf_	ΔG_gas_	ΔG_solv_	ΔG_total_
Curcumin	−38.18 ± 3.20	−25.84 ± 3.43	42.16 ± 2.29	−5.21 ± 0.31	−64.02 ± 4.12	36.96 ± 2.15	−27.06 ± 3.22
**B5**	−73.18 ± 3.97	−33.57 ± 7.04	61.54 ± 8.20	−9.81 ± 0.52	−106.76 ± 9.26	51.74 ± 7.89	−55.02 ± 3.22

**E_vdw_** represents the van der Waals energy, **E_el_** represents the electrostatic energy, **E_gb_** represents the polar solvation energy, **E_surf_** represents the nonpolar solvation energy. **ΔG_gas_** represents the gas-phase energy, **ΔG_gas_** = **E_vdw_** + **E_el_**, **ΔG_solv_** represents solvation free energy, **ΔG_solv_** = **E_gb_** + **E_surf_**, **ΔG_total_** represents total free energy, **ΔG_total_** = **ΔG_gas_** + **ΔG_solv_**.

**Table 3:** Key residues of curcumin, B5 with AKT by decomposition free energy calculations.

Compounds	Key Residues (<−1.0 Kcal/mol)
Curcumin	LEU156, VAL164, THR211, MET227, GLU228, TYR229, THR291, PHE442
**B5**	LEU156, GLY157, PHE161, LYS163, VAL164, HIE194, MET281, LEU295, PHE309, CYS310, PRO313, LEU316

## Data Availability

Data is contained within the article and supplementary materials.

## Supplementary Materials

The following supporting information can be downloaded at: https://www.mdpi.com/article/10.3390/ph15080950/s1, Figure S1: ^1^H NMR, ^13^C NMR of target compounds **A1**–**A8**, **B1**–**B7**, **C1**–**C6**.
