# Supplementary material for: Design, Synthesis, and Antitumor Evaluation of Novel Mono-Carbonyl Curcumin Analogs in Hepatocellular Carcinoma Cell
Source: Pharmaceuticals (Basel). 2022 Jul 30;15(8):950. doi: 10.3390/ph15080950 (PMC9413278; doi:10.3390/ph15080950)
Supplement: Supplementary file 1 [file pharmaceuticals-15-00950-s001.zip › pharmaceuticals-1800068-supplementary.pdf]

Design, synthesis and antitumor evaluation of novel mono-carbonyl curcumin analogs in hepatocellular carcinoma cell

Pan Yu[a, 1, *], Weiya Cao[a, 1], Linguo Zhao[b,*], Qing Han[c], Shilong Yang[b], Kepeng Yang[a], Xiaolei Pan[a], Qianyun Wang[a], Yuan Wang[a]

a College of Medicine, Anhui University of Science and Technology, Huainan, 232001, China

b College of Chemical Engineering, Nanjing Forestry University, Nanjing, 210037, China

c College of Science, China Agricultural University, Beijing, 100193, China

1 These authors equally contributed to this work.

* Correspondence: 2020035@aust.edu.cn (Pan Yu); njfu2304@163.com (Linguo Zhao).

^{1}H NMR, ^{13}C NMR of target compounds A1-A8

((1E,6E)-3,5-dioxohepta-1,6-diene-1,7-diyl)bis(2-methoxy-4,1-phenylene) (2E,2'E)-bis(3-(4-fluorophenyl)acrylate) (**A1**)

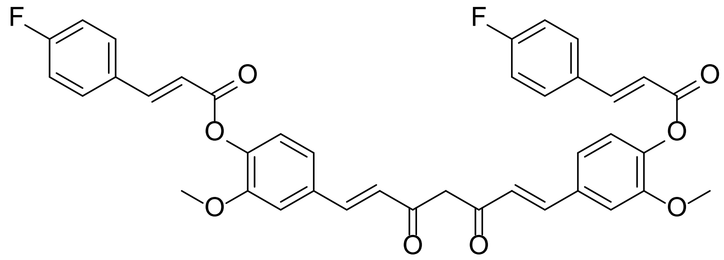

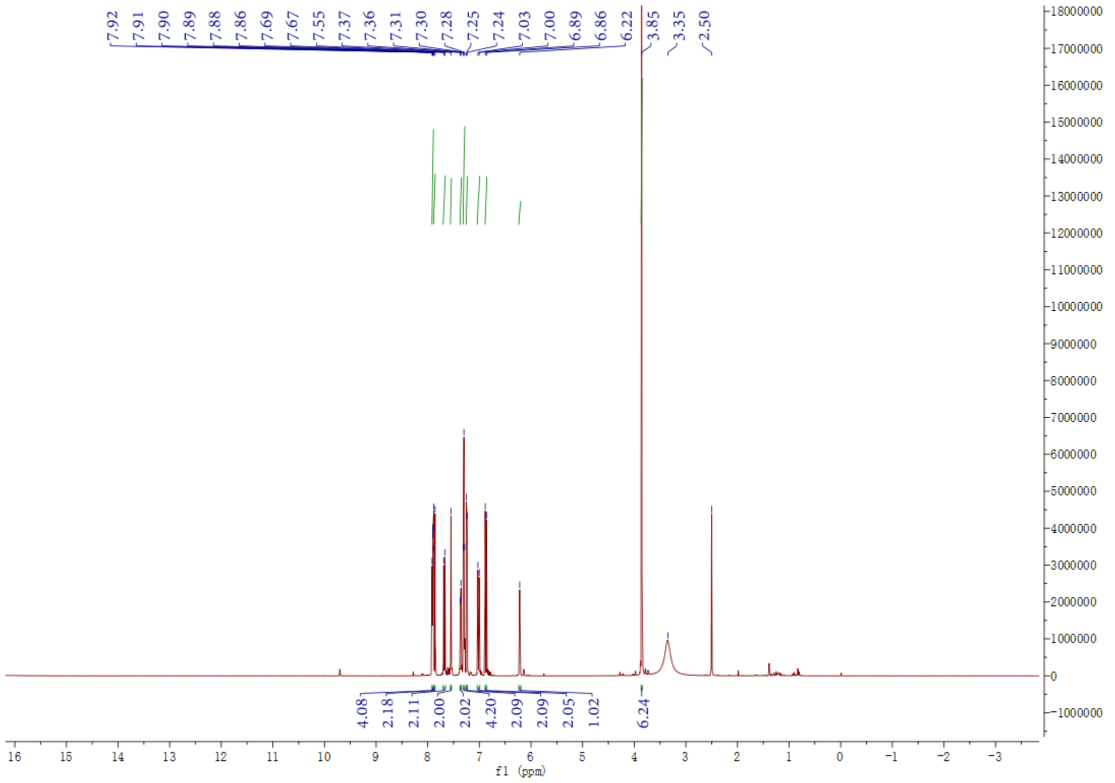

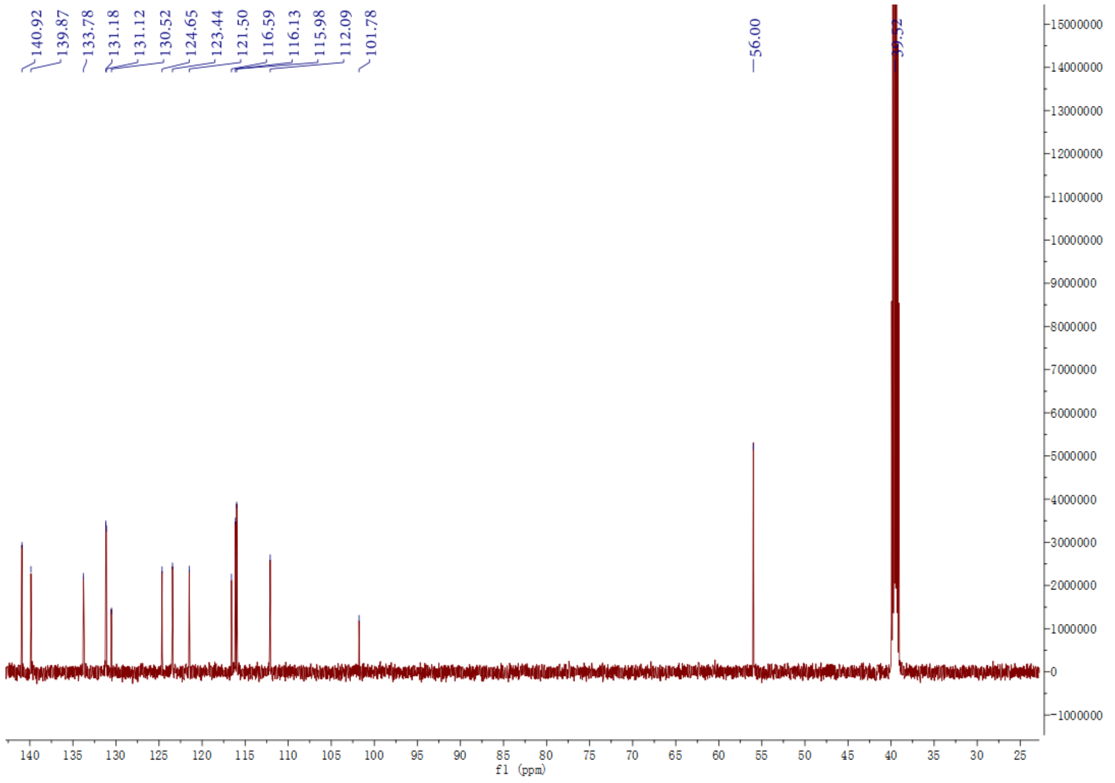

((1E,6E)-3,5-dioxohepta-1,6-diene-1,7-diyl)bis(2-methoxy-4,1-phenylene) (2E,2'E)-bis(3-(2-chlorophenyl)acrylate) (**A2**)

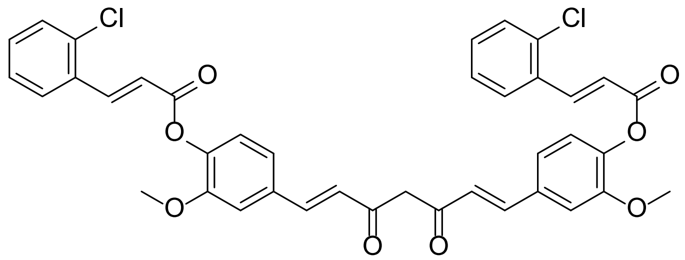

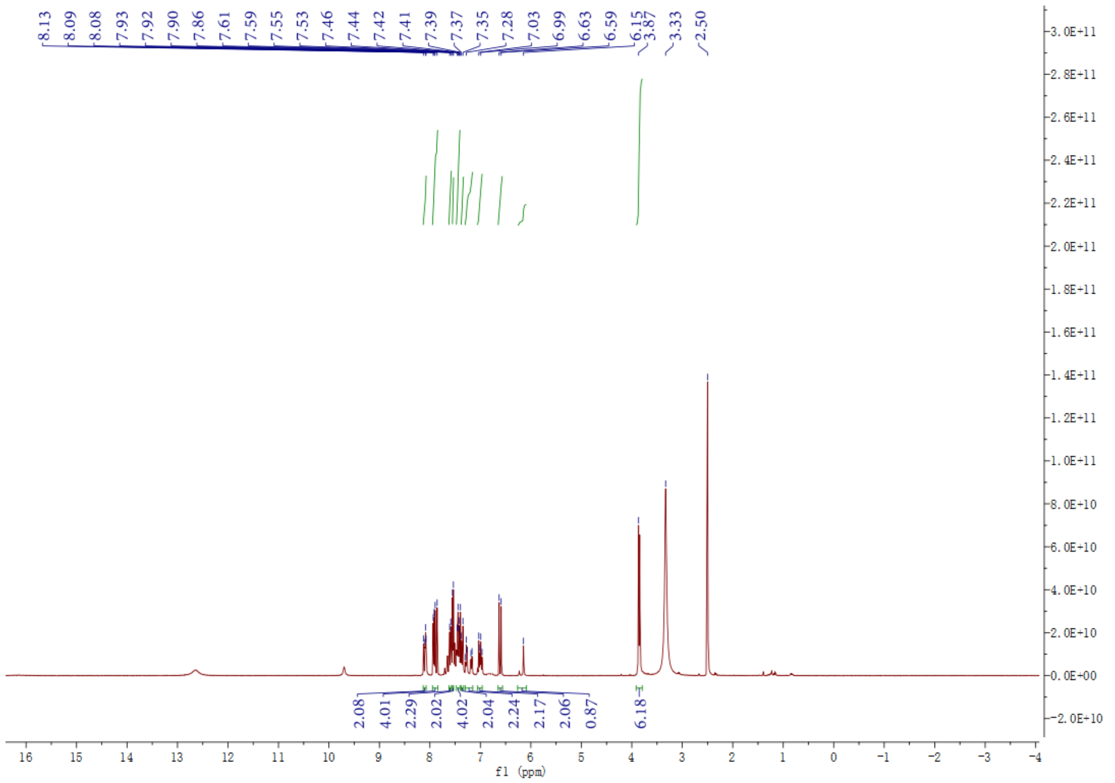

((1E,6E)-3,5-dioxohepta-1,6-diene-1,7-diyl)bis(2-methoxy-4,1-phenylene) (2E,2'E)-bis(3-(3-chlorophenyl)acrylate) (**A3**)

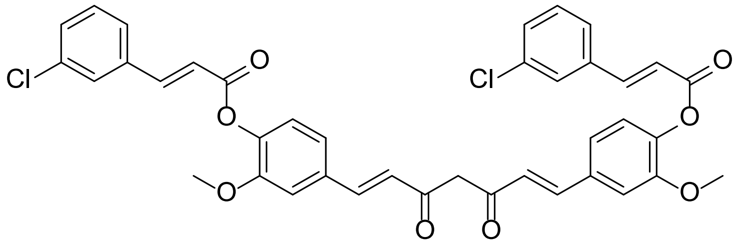

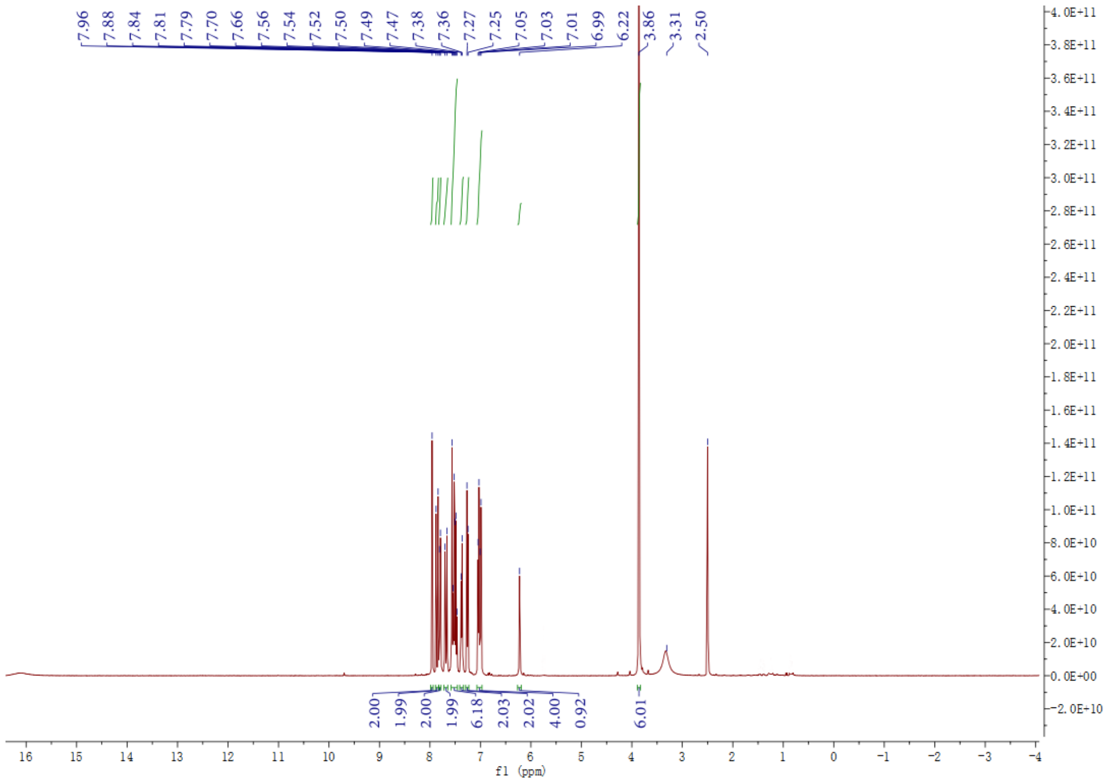

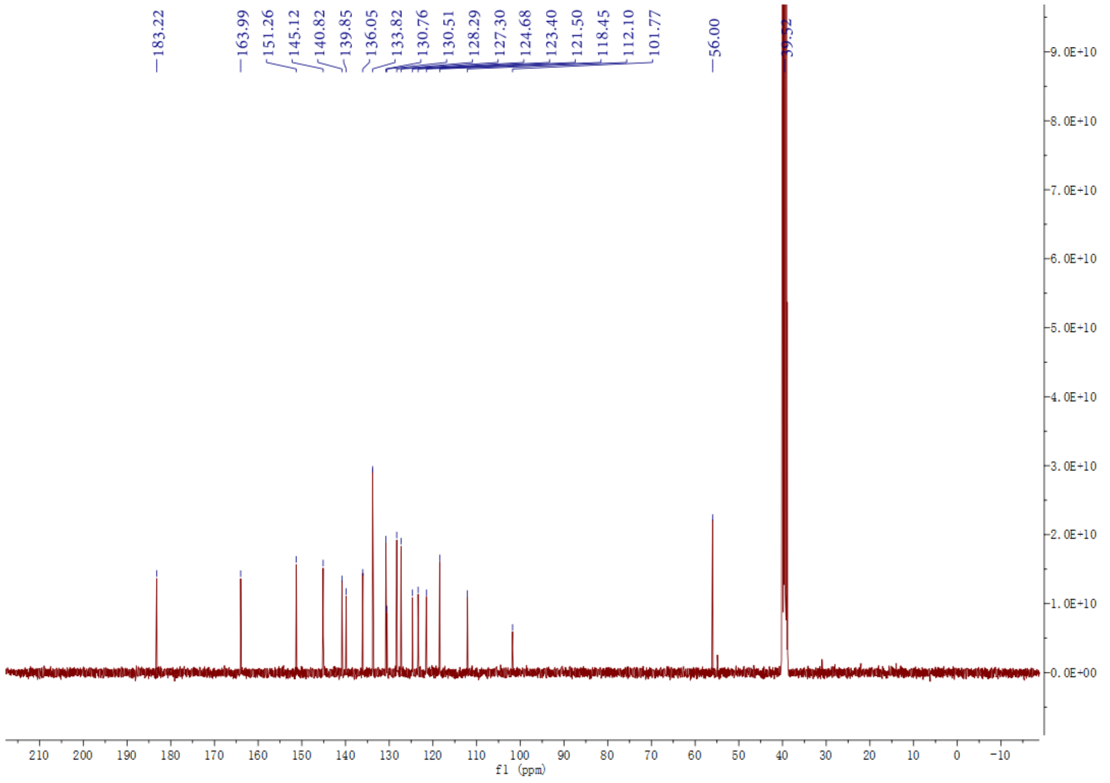

((1E,6E)-3,5-dioxohepta-1,6-diene-1,7-diyl)bis(2-methoxy-4,1-phenylene) (2E,2'E)-bis(3-(3-(trifluoromethyl)phenyl)acrylate) (**A4**)

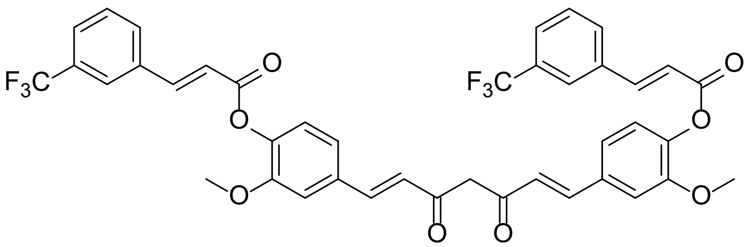
F3C
O
O
F3C
O
O
O
O
O
O

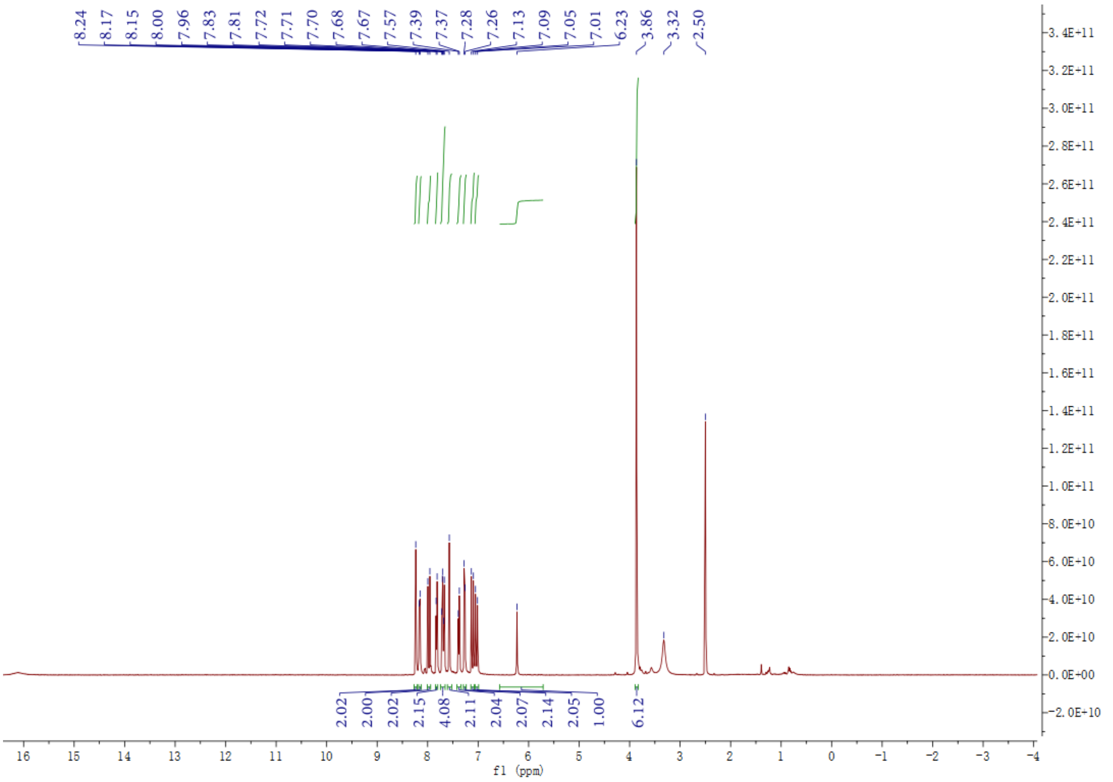
8.24
8.17
8.15
8.00
7.96
7.83
7.81
7.72
7.71
7.70
7.68
7.67
7.57
7.39
7.37
7.28
7.26
7.13
7.09
7.05
7.01
6.23
3.86
3.32
2.50
2.02
2.00
2.02
2.15
4.08
2.11
2.04
2.07
2.14
2.05
1.00
6.12
f1 (ppm)

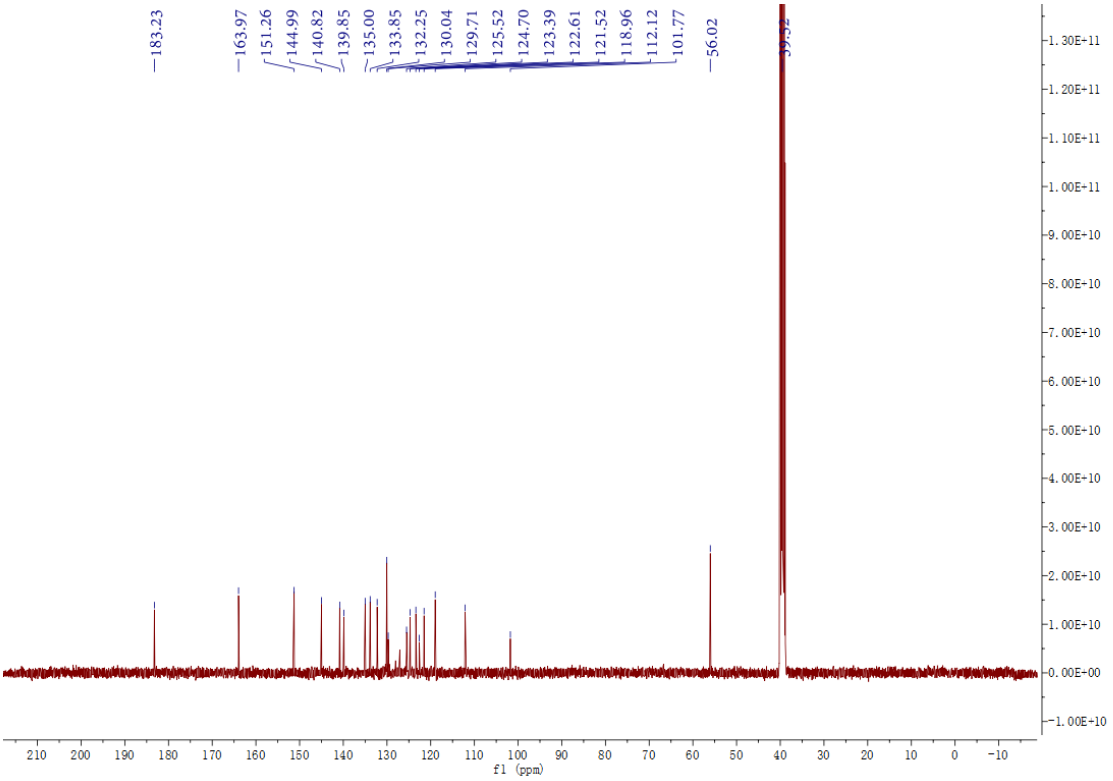
183.23
163.97
151.26
144.99
140.82
139.85
135.00
133.85
132.25
130.04
129.71
125.52
124.70
123.39
122.61
121.52
118.96
112.12
101.77
56.02
39.52
f1 (ppm)

((1E,6E)-3,5-dioxohepta-1,6-diene-1,7-diyl)bis(2-methoxy-4,1-phenylene) (2E,2'E)-bis(3-(4-(trifluoromethyl)phenyl)acrylate) (**A5**)

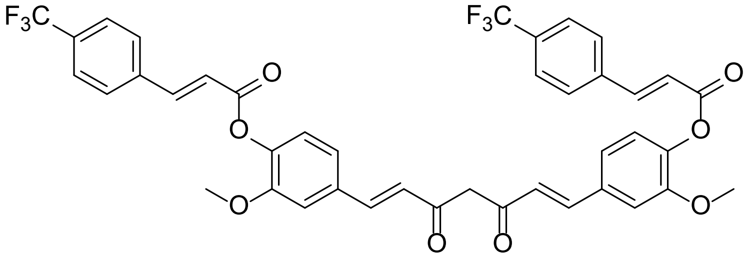

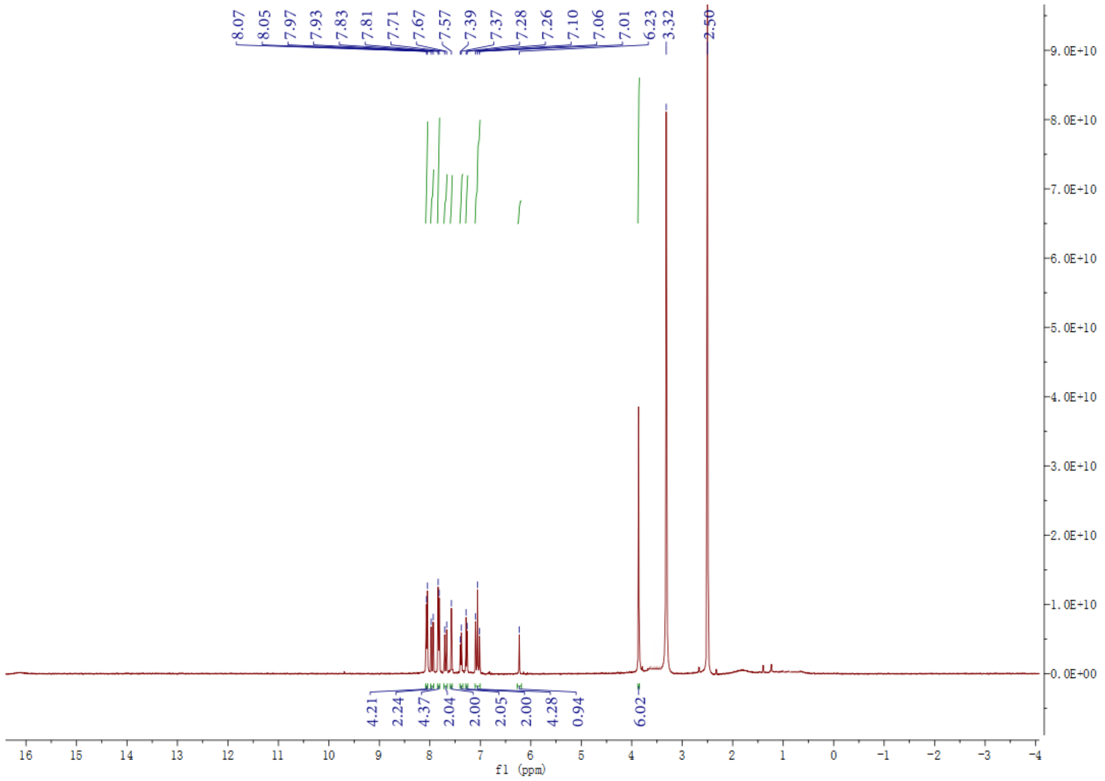

((1E,6E)-3,5-dioxohepta-1,6-diene-1,7-diyl)bis(2-methoxy-4,1-phenylene) (2E,2'E)-bis(3-(3-nitrophenyl)acrylate) (**A6**)

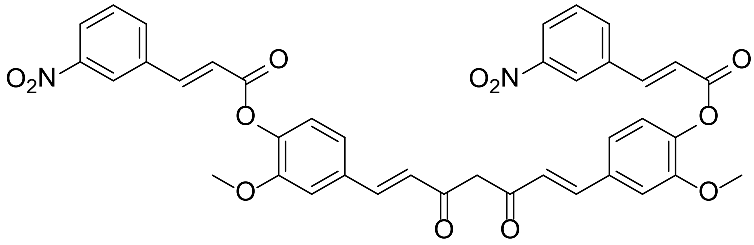

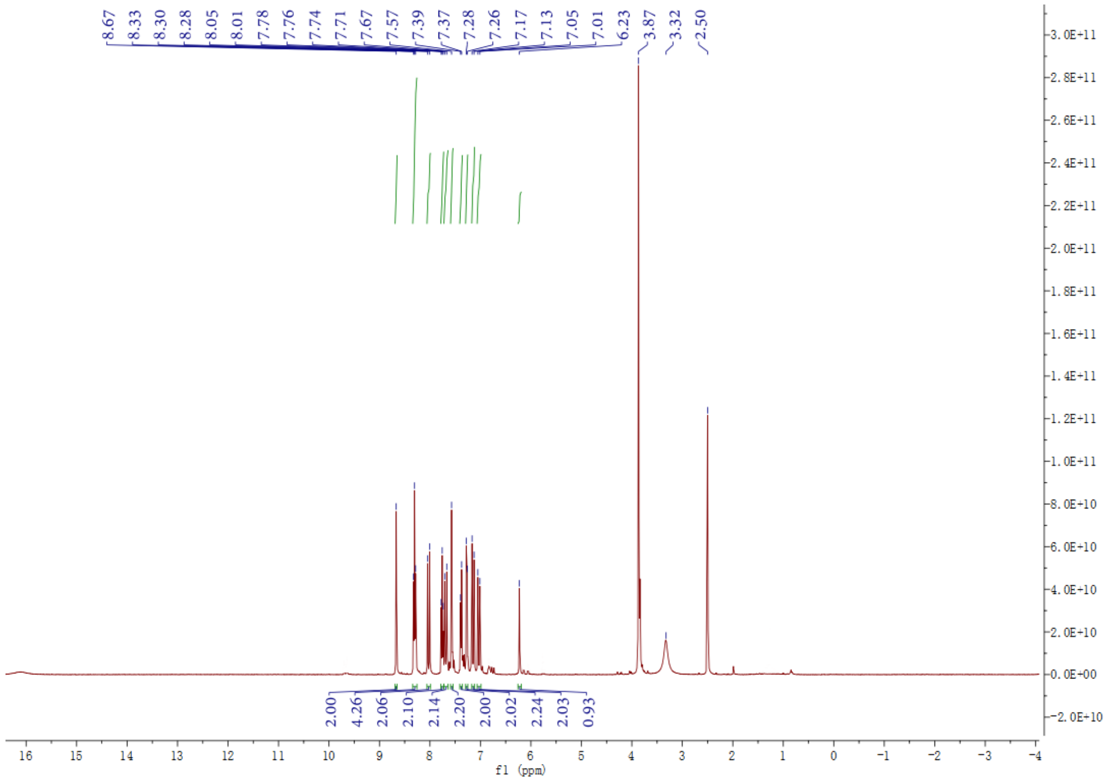

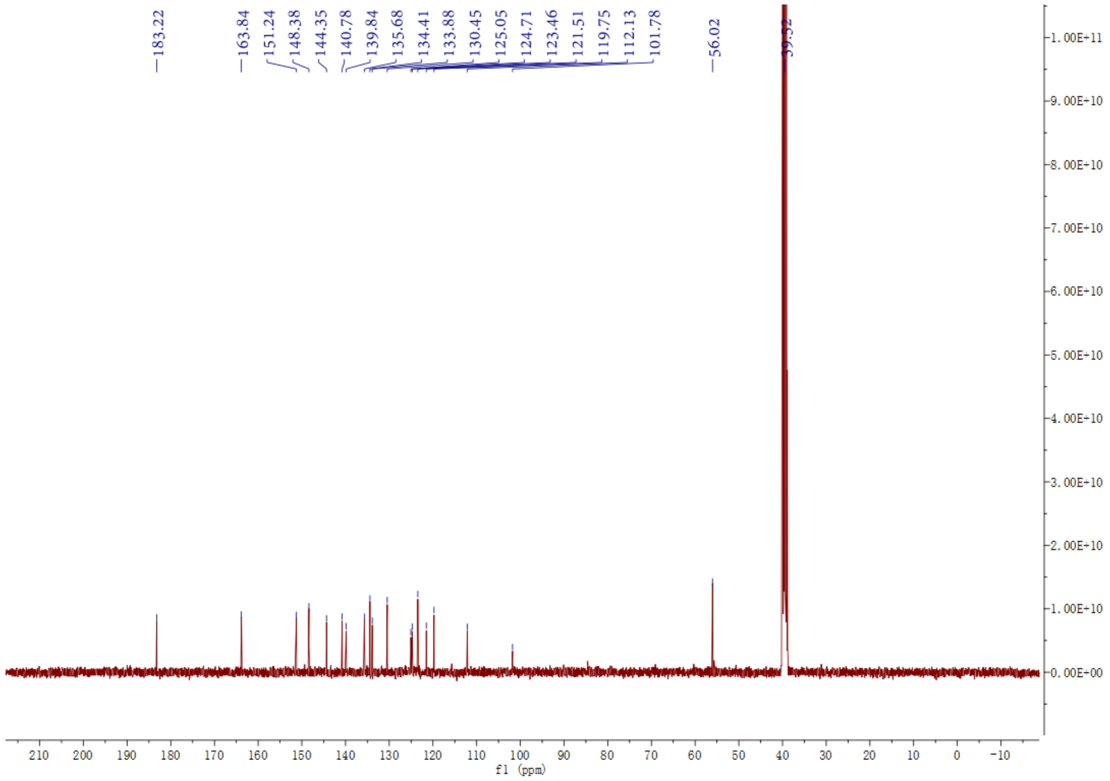

((1E,6E)-3,5-dioxohepta-1,6-diene-1,7-diyl)bis(2-methoxy-4,1-phenylene) (2E,2'E)-bis(3-(2-nitrophenyl)acrylate) (**A7**)

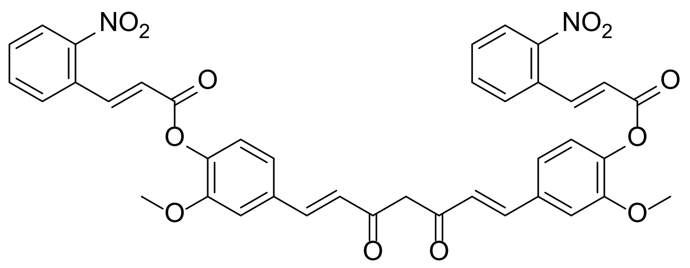

NO2
O
O
O
O
O
O
NO2
O
O
O

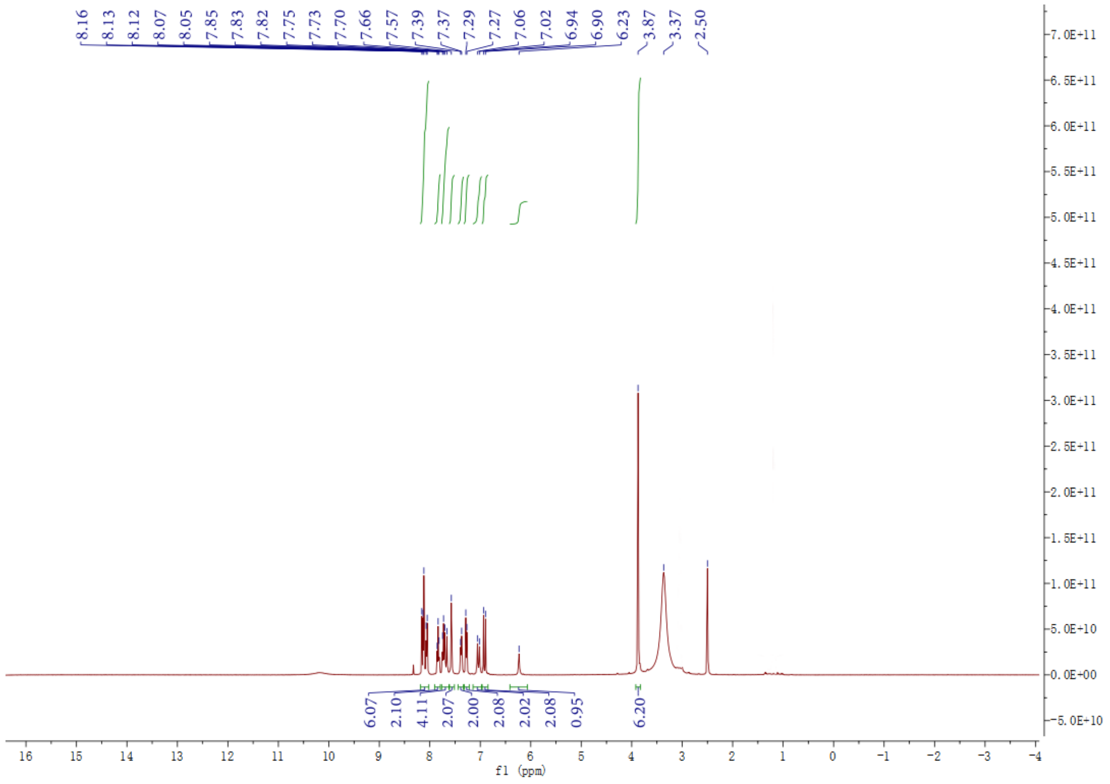

8.16
8.13
8.12
8.07
8.05
7.85
7.83
7.82
7.75
7.73
7.70
7.66
7.57
7.39
7.37
7.29
7.27
7.06
7.02
6.94
6.90
6.23
3.87
3.37
2.50
6.07
2.10
4.11
2.07
2.00
2.08
2.02
2.08
0.95
6.20
f1 (ppm)

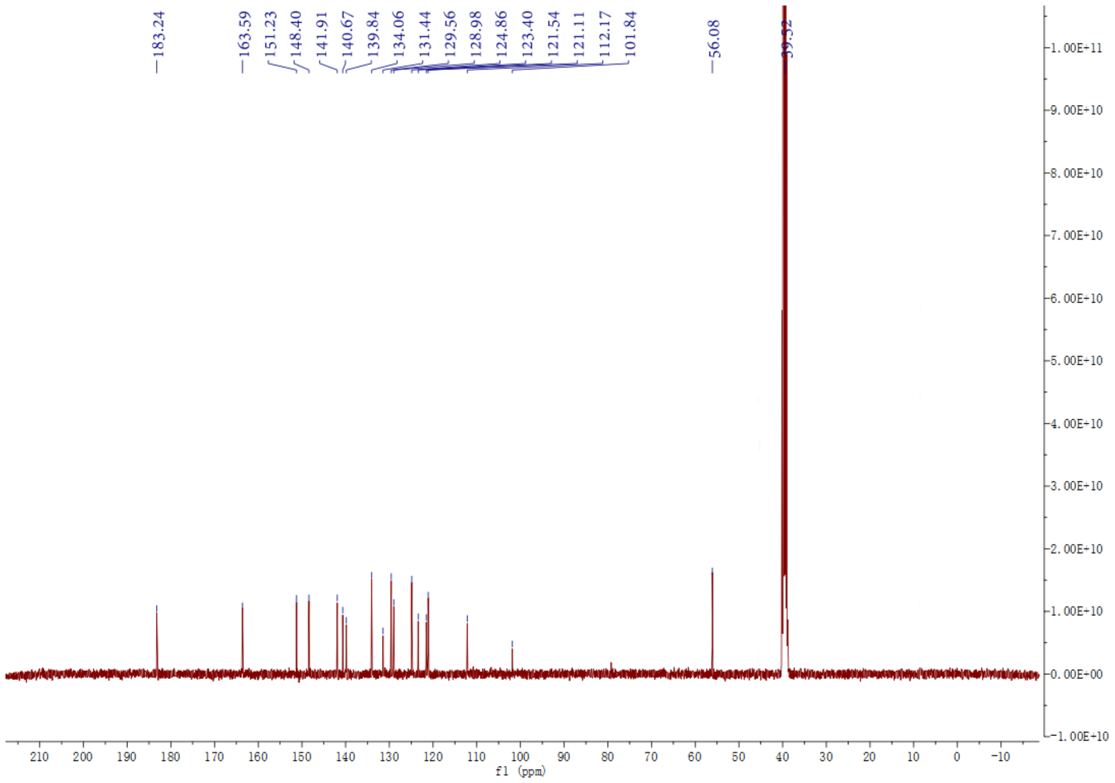

((1E,6E)-3,5-dioxohepta-1,6-diene-1,7-diyl)bis(2-methoxy-4,1-phenylene) (2E,2'E)-bis(3-(p-tolyl)acrylate) (**A8**)

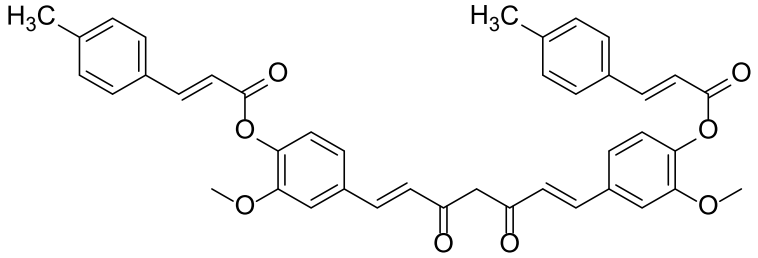

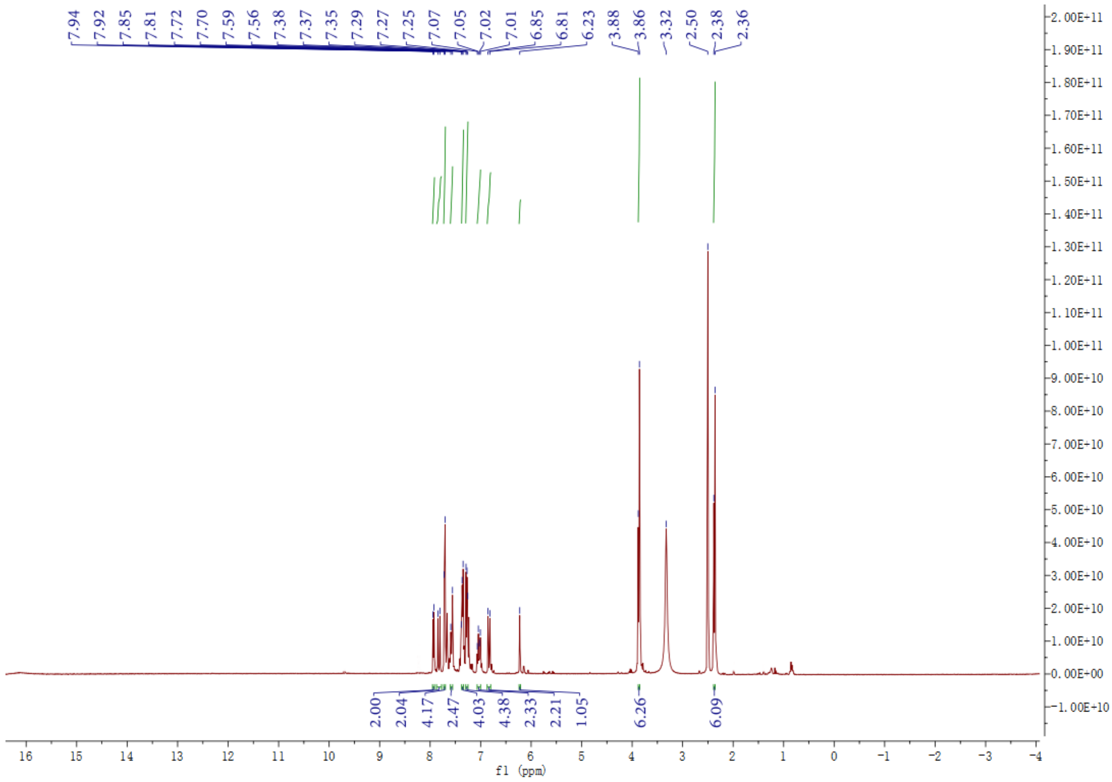

^{1}H NMR, ^{13}C NMR of target compounds B1-B7

(1E,4E)-1,5-bis(4-hydroxy-3-methoxyphenyl)penta-1,4-dien-3-one (**3**)

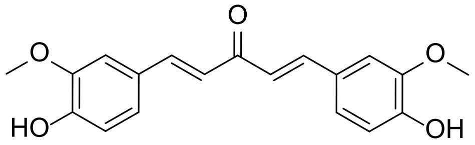

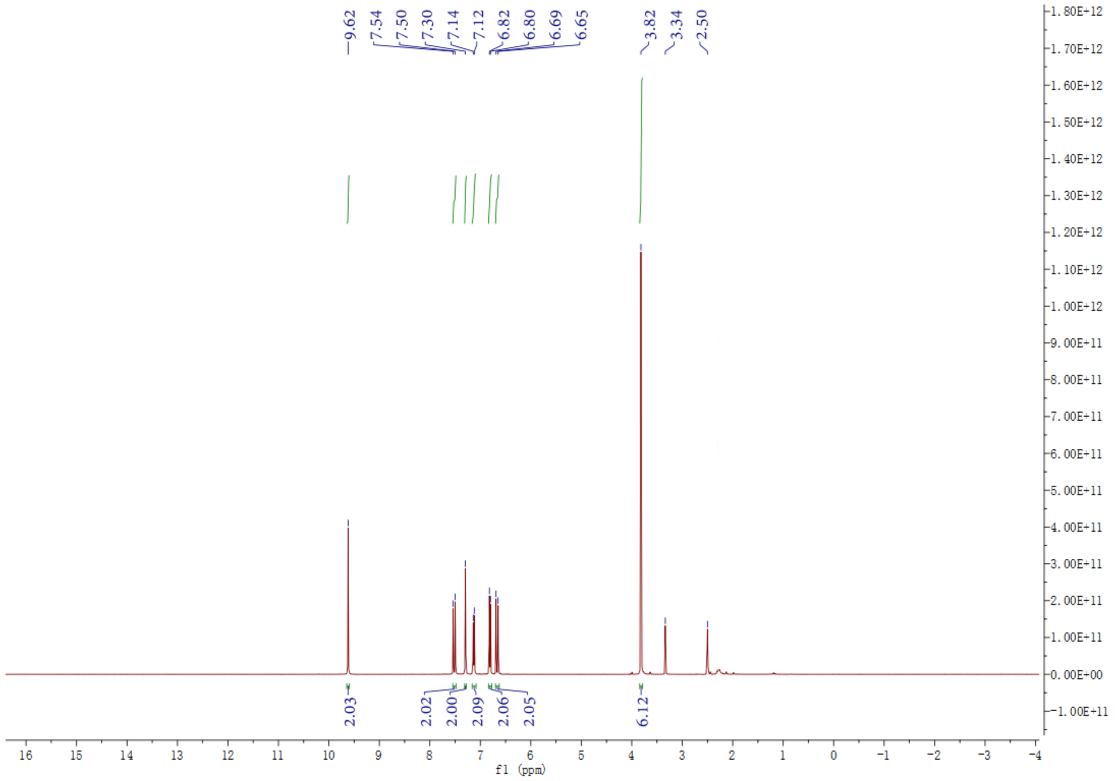

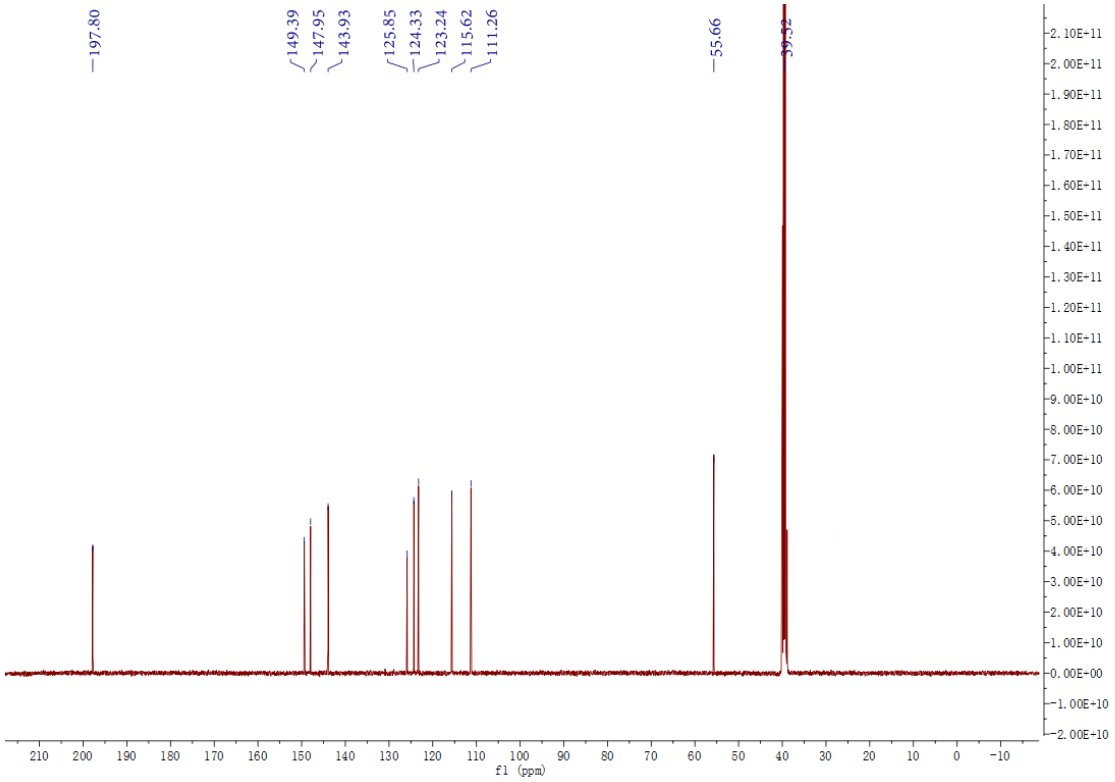

((1E,4E)-3-oxopenta-1,4-diene-1,5-diyl)bis(2-methoxy-4,1-phenylene) (2E,2'E)-bis(3-(4-fluorophenyl)acrylate) (**B1**)

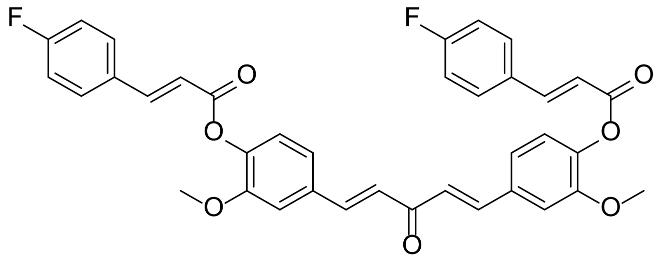

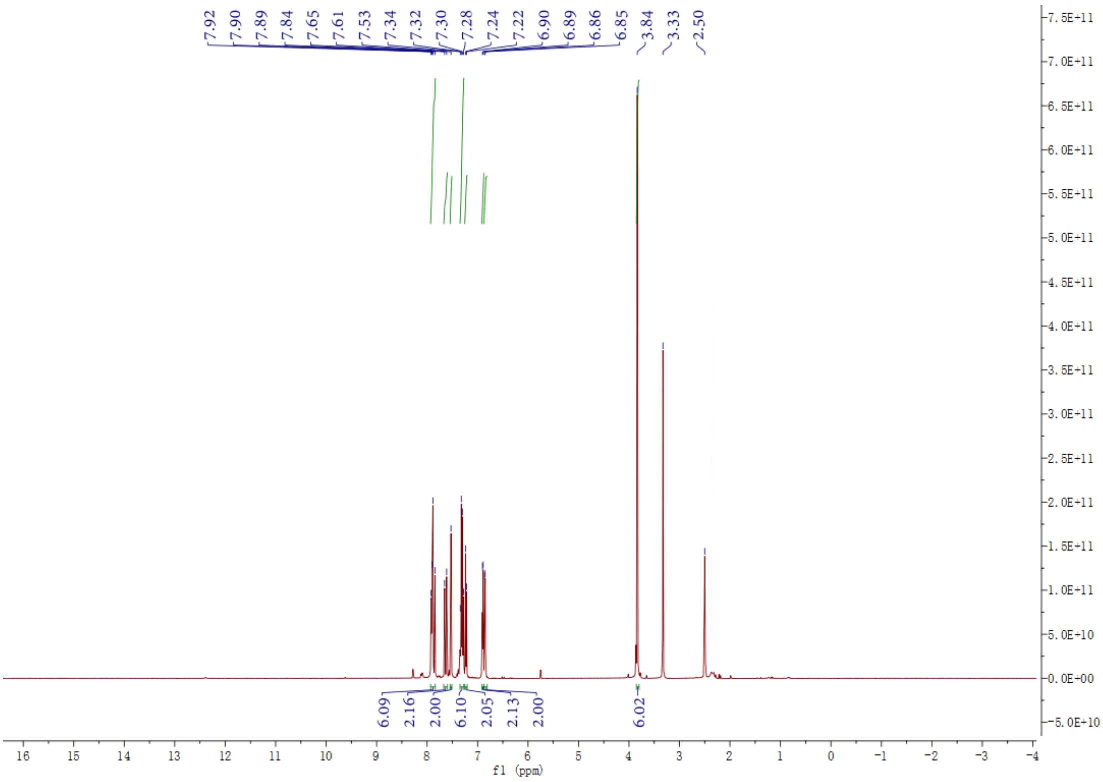
7.92
7.90
7.89
7.84
7.65
7.61
7.53
7.34
7.32
7.30
7.28
7.24
7.22
6.90
6.89
6.86
6.85
3.84
3.33
2.50
6.09
2.16
2.00
6.10
2.05
2.13
2.00
6.02
f1 (ppm)

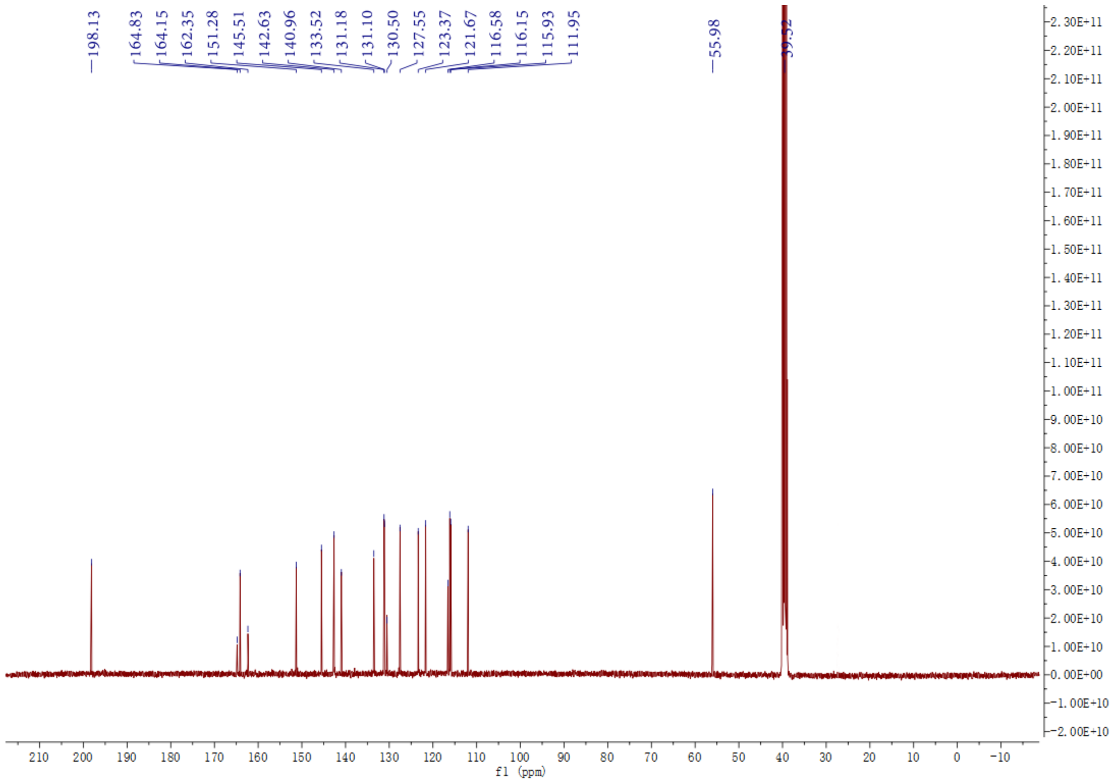

((1E,4E)-3-oxopenta-1,4-diene-1,5-diyl)bis(2-methoxy-4,1-phenylene) (2E,2'E)-bis(3-(2-chlorophenyl)acrylate) (**B2**)

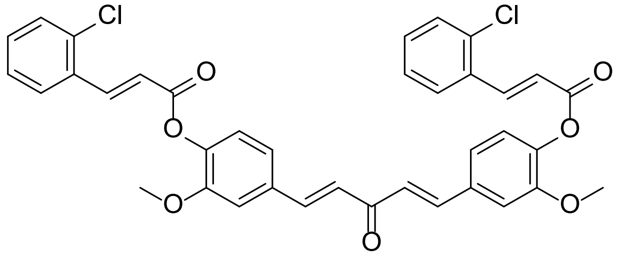

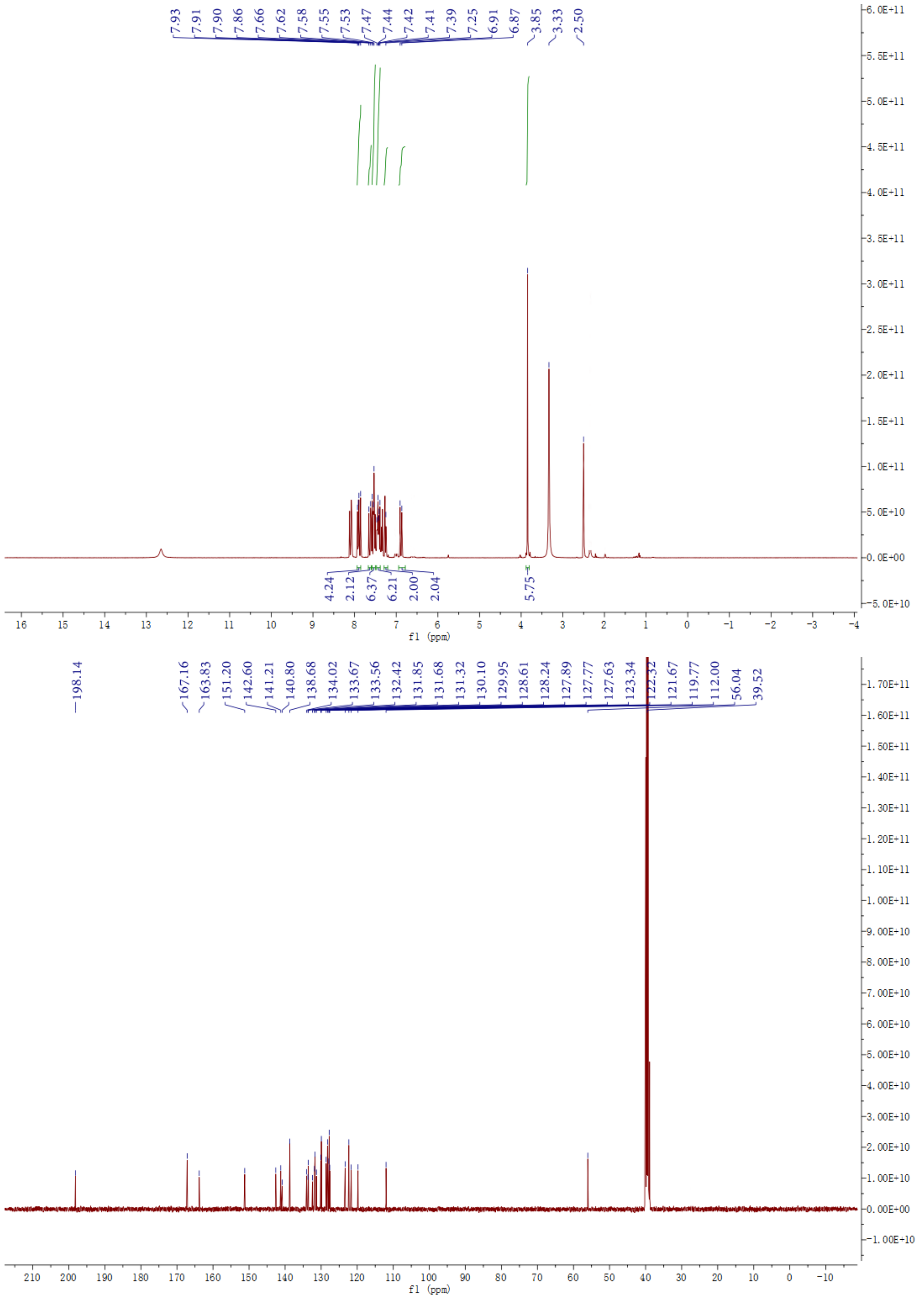

((1E,4E)-3-oxopenta-1,4-diene-1,5-diyl)bis(2-methoxy-4,1-phenylene) (2E,2'E)-bis(3-(2-fluorophenyl)acrylate) (**B3**)

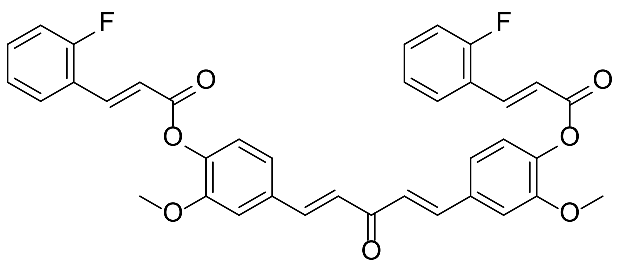

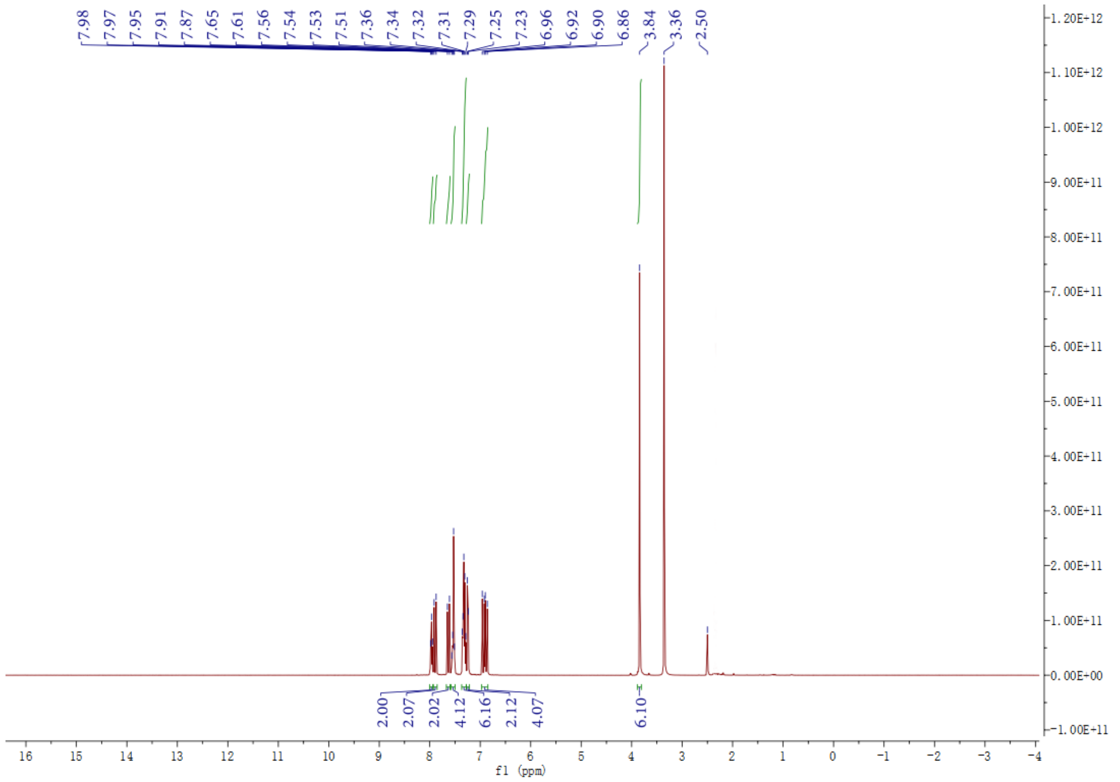

((1E,4E)-3-oxopenta-1,4-diene-1,5-diyl)bis(2-methoxy-4,1-phenylene) (2E,2'E)-bis(3-(3-(trifluoromethyl)phenyl)acrylate) (**B4**)

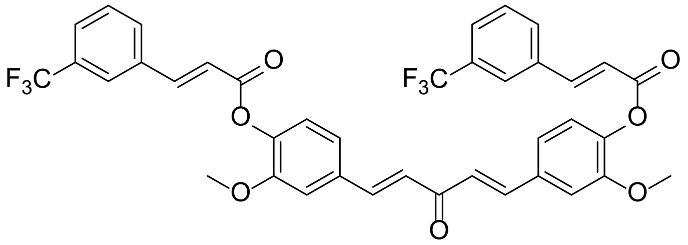

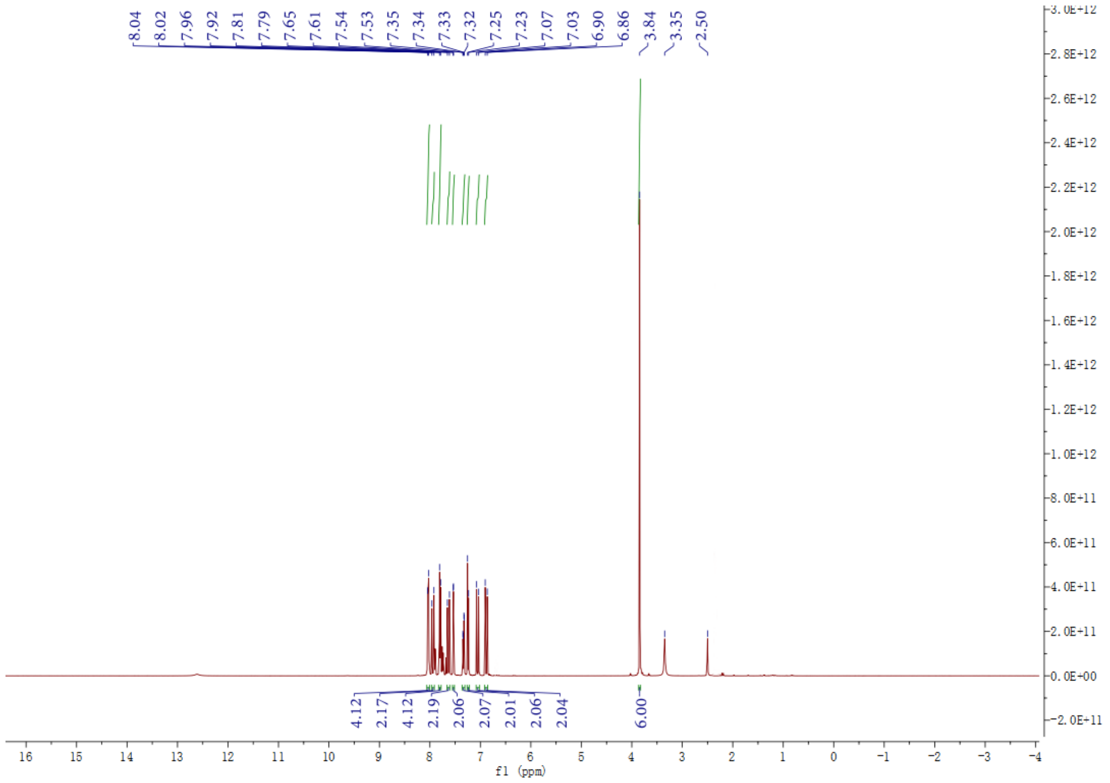

((1E,4E)-3-oxopenta-1,4-diene-1,5-diyl)bis(2-methoxy-4,1-phenylene) (2E,2'E)-bis(3-(3-nitrophenyl)acrylate) (**B5**)

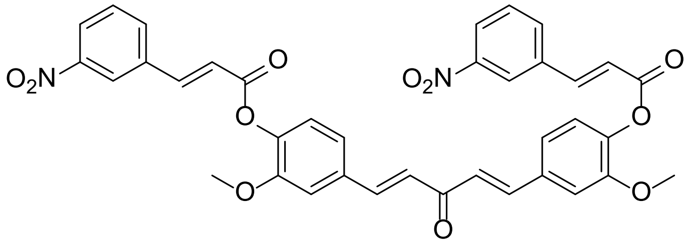

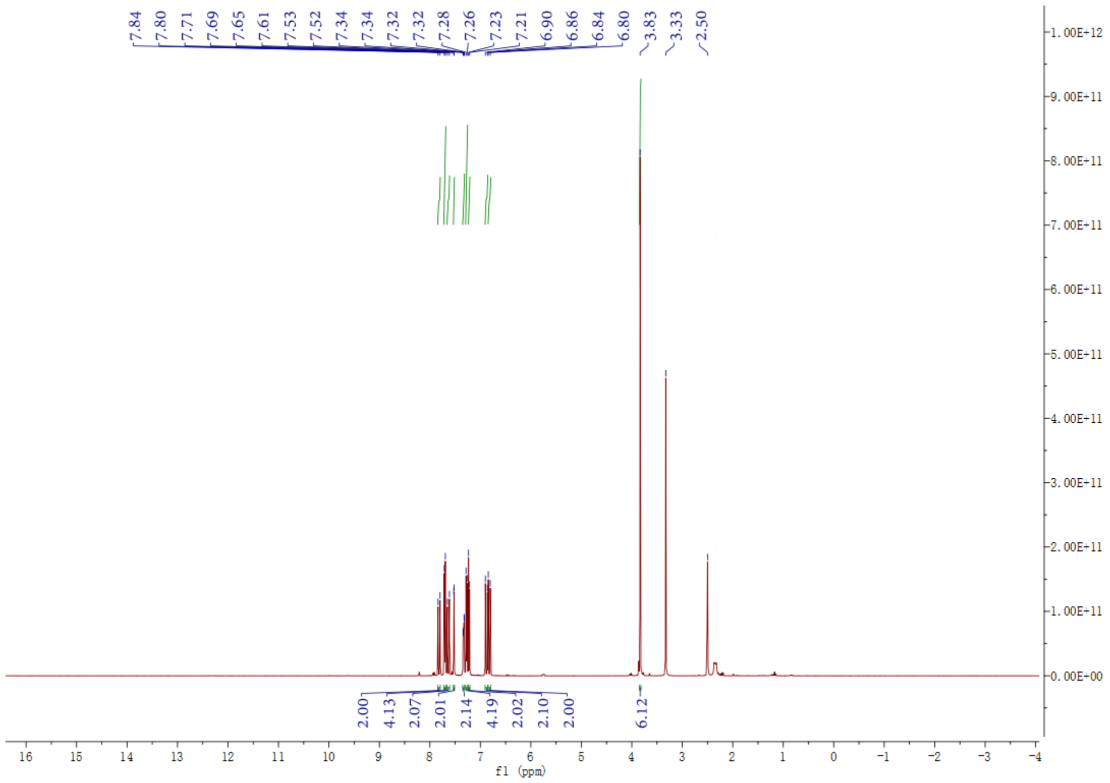

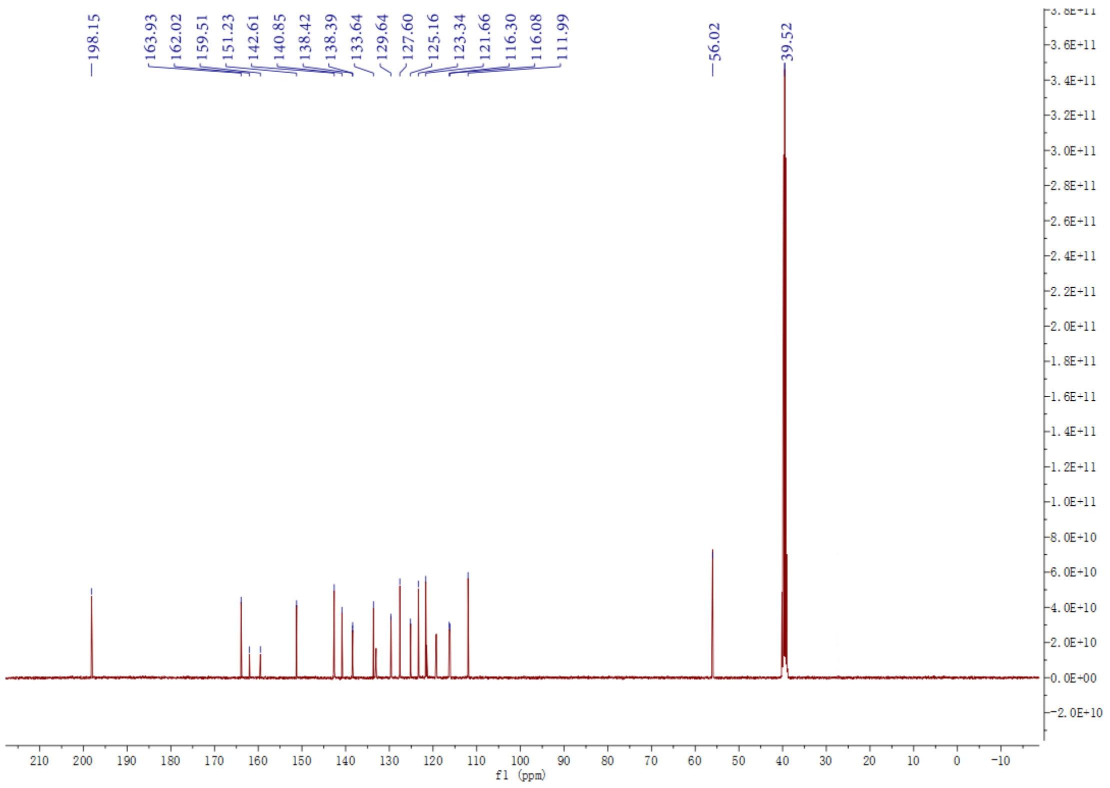

((1E,4E)-3-oxopenta-1,4-diene-1,5-diyl)bis(2-methoxy-4,1-phenylene) (2E,2'E)-bis(3-(4-nitrophenyl)acrylate) (**B6**)

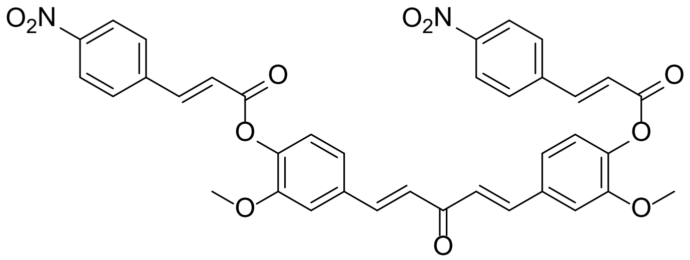

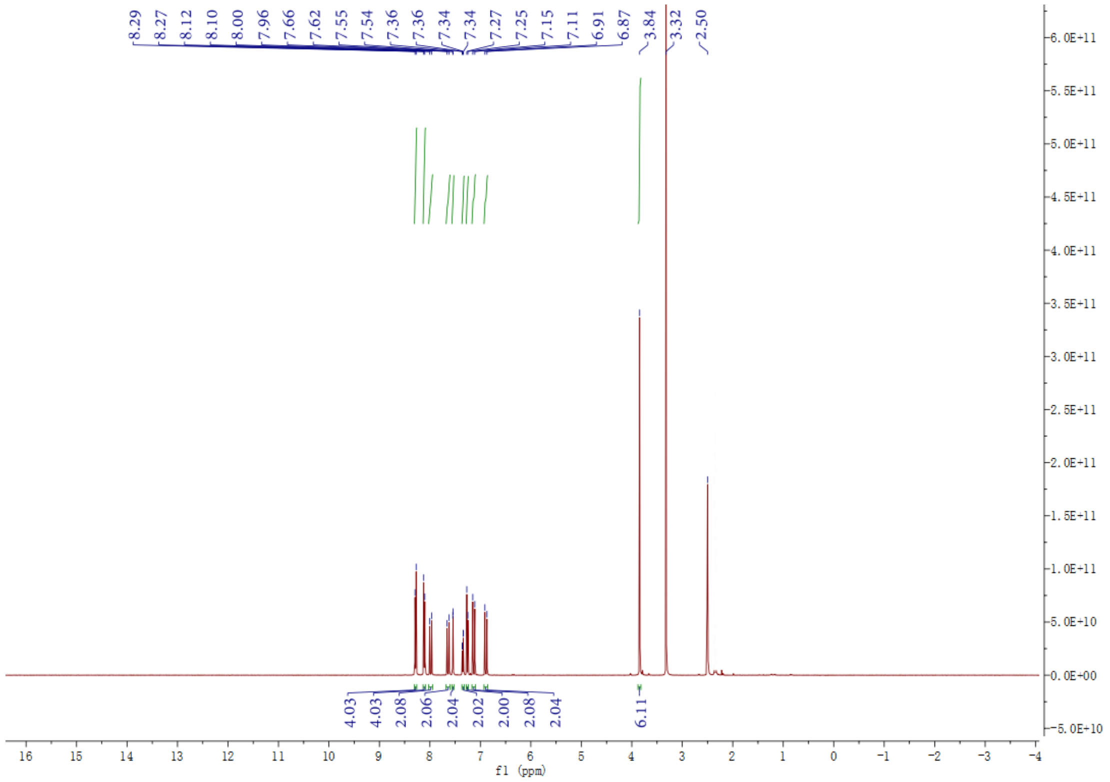

((1E,4E)-3-oxopenta-1,4-diene-1,5-diyl)bis(2-methoxy-4,1-phenylene) (2E,2'E)-bis(3-(p-tolyl)acrylate) (**B7**)

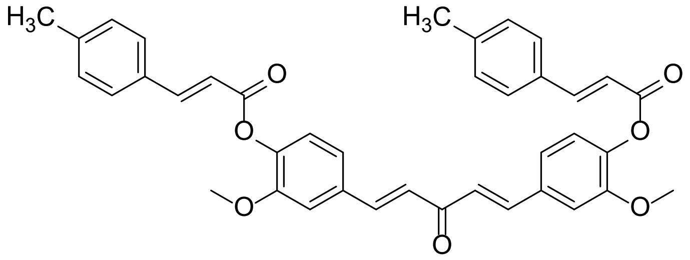

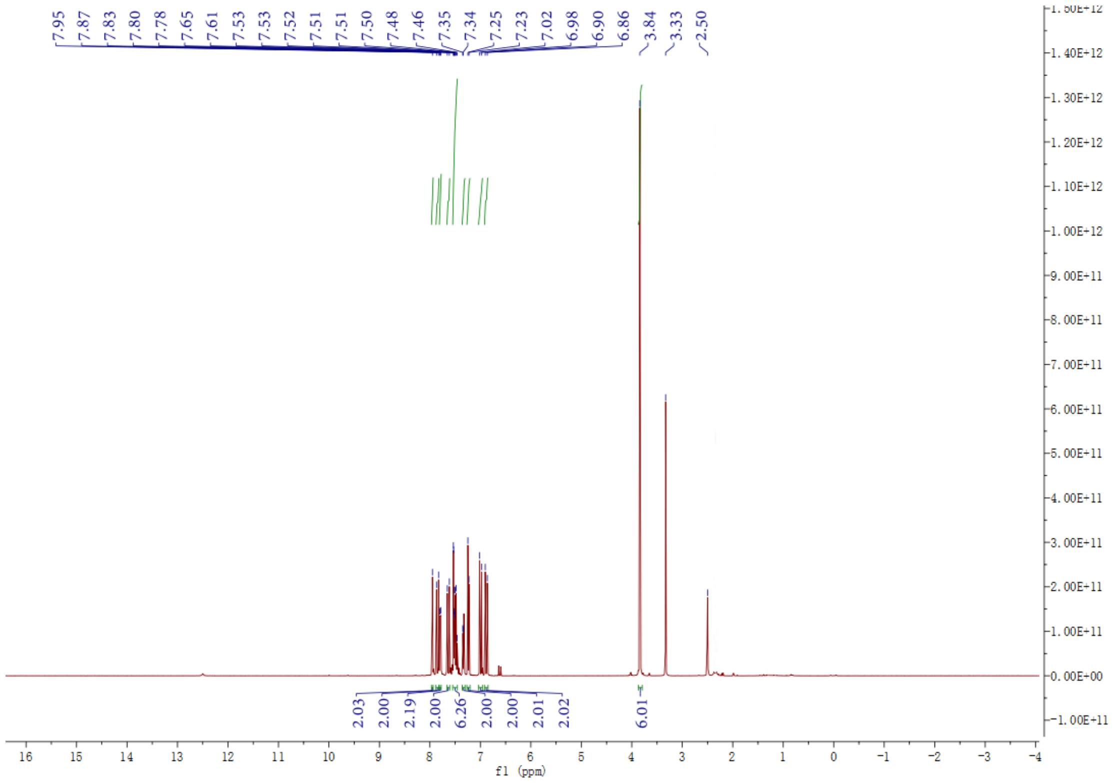

^{1}H NMR, ^{13}C NMR of target compounds C1-C6

(E)-1,3-bis(4-hydroxy-3-methoxyphenyl)prop-2-en-1-one (**8**)

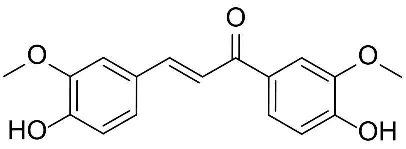

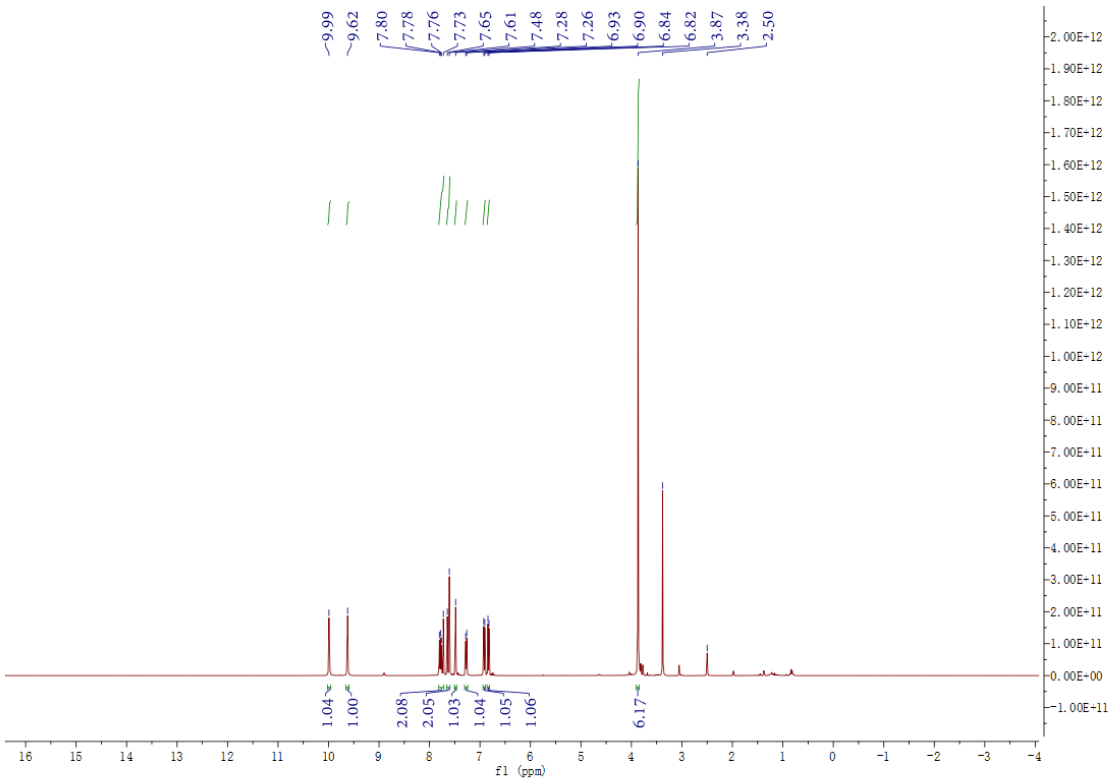

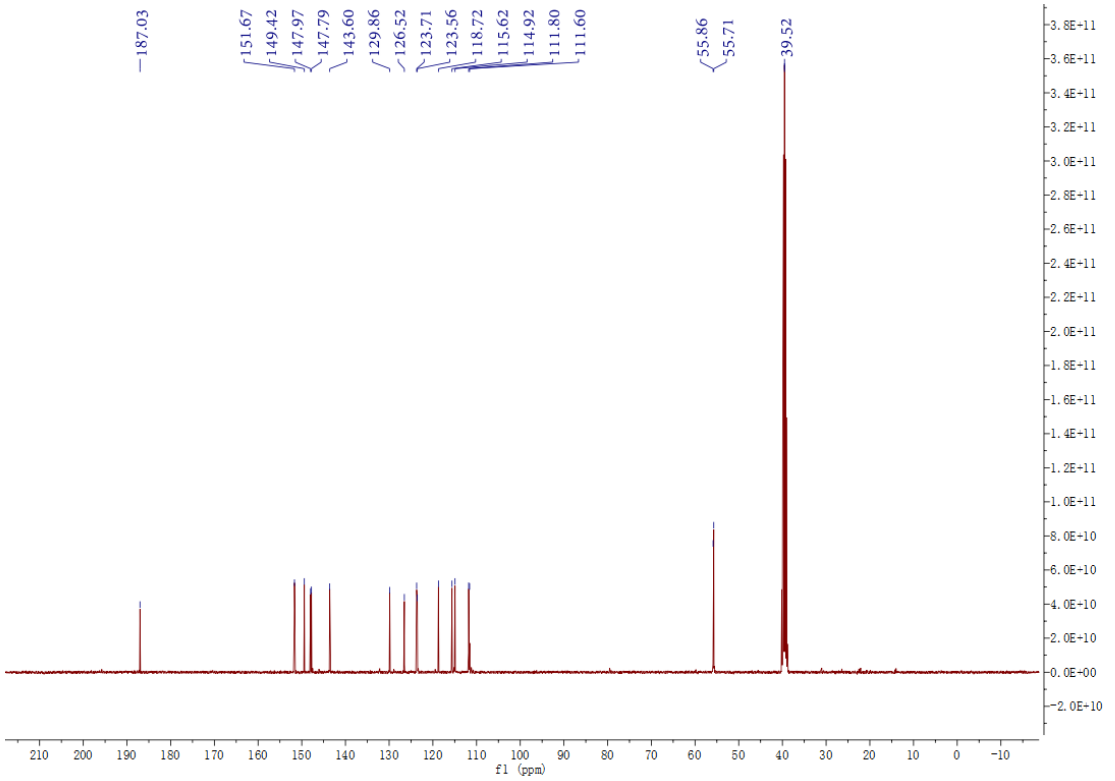

((E)-3-oxoprop-1-ene-1,3-diyl)bis(2-methoxy-4,1-phenylene) (2E,2'E)-bis(3-(4-fluorophenyl)acrylate) (**C1**)

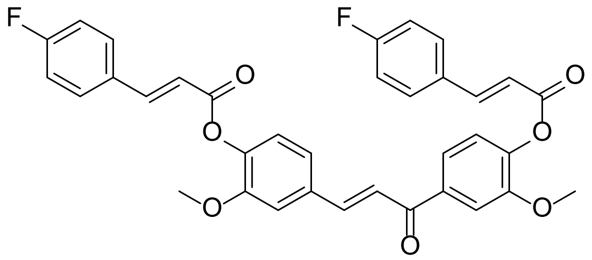

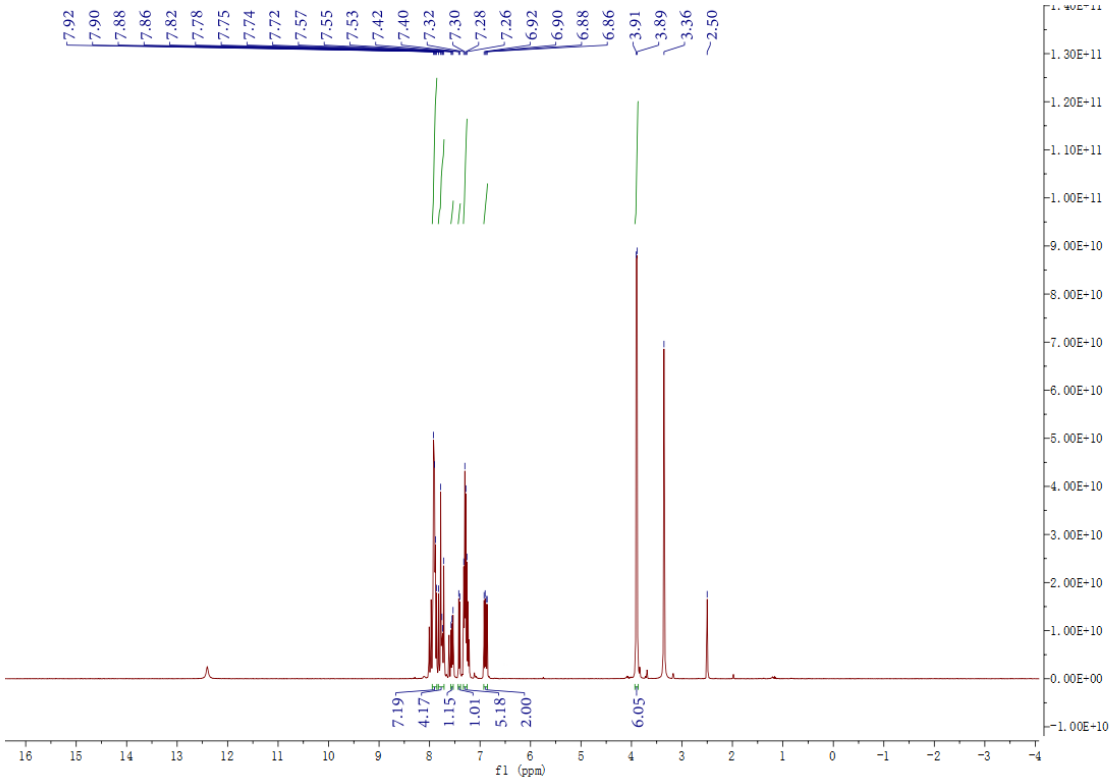
7.92 7.90 7.88 7.86 7.82 7.78 7.75 7.74 7.72 7.57 7.55 7.53 7.42 7.40 7.32 7.30 7.28 7.26 6.92 6.90 6.88 6.86 3.91 3.89 3.36 2.50
7.19 4.17 1.15 1.01 5.18 2.00 6.05
f1 (ppm)

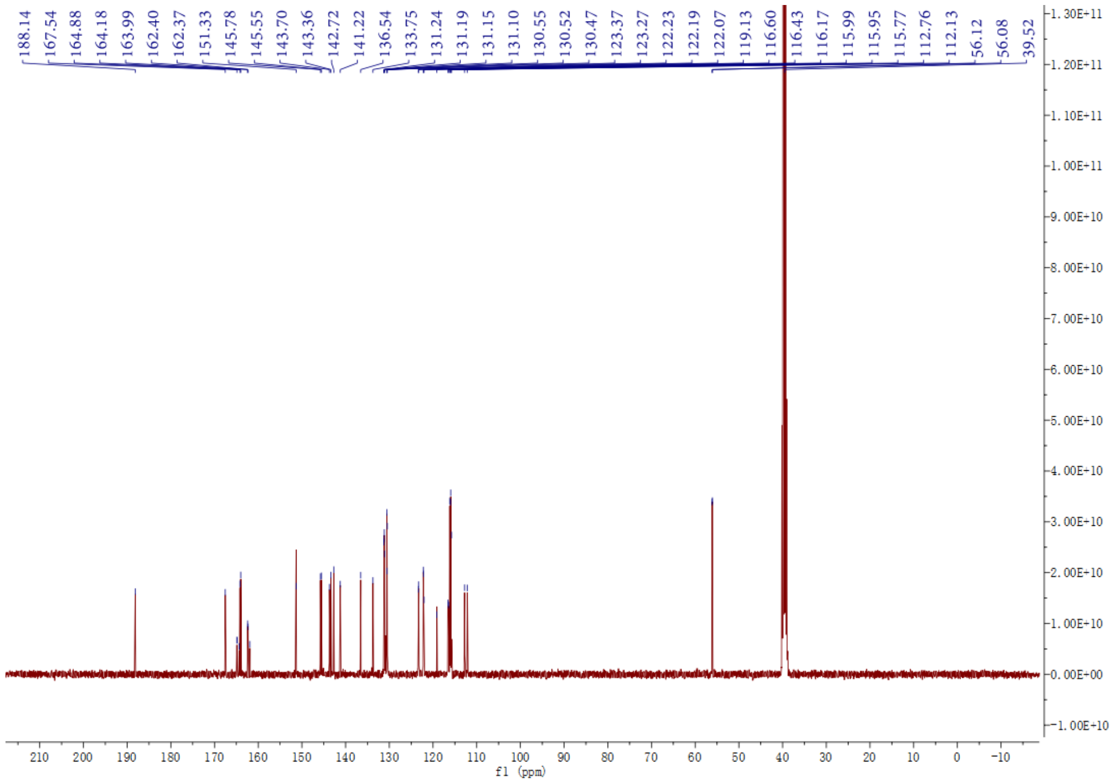

((E)-3-oxoprop-1-ene-1,3-diyl)bis(2-methoxy-4,1-phenylene) (2E,2'E)-bis(3-(2-chlorophenyl)acrylate) (**C2**)

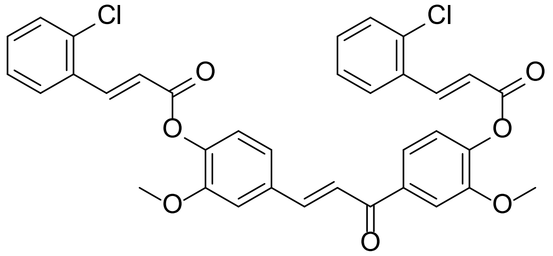

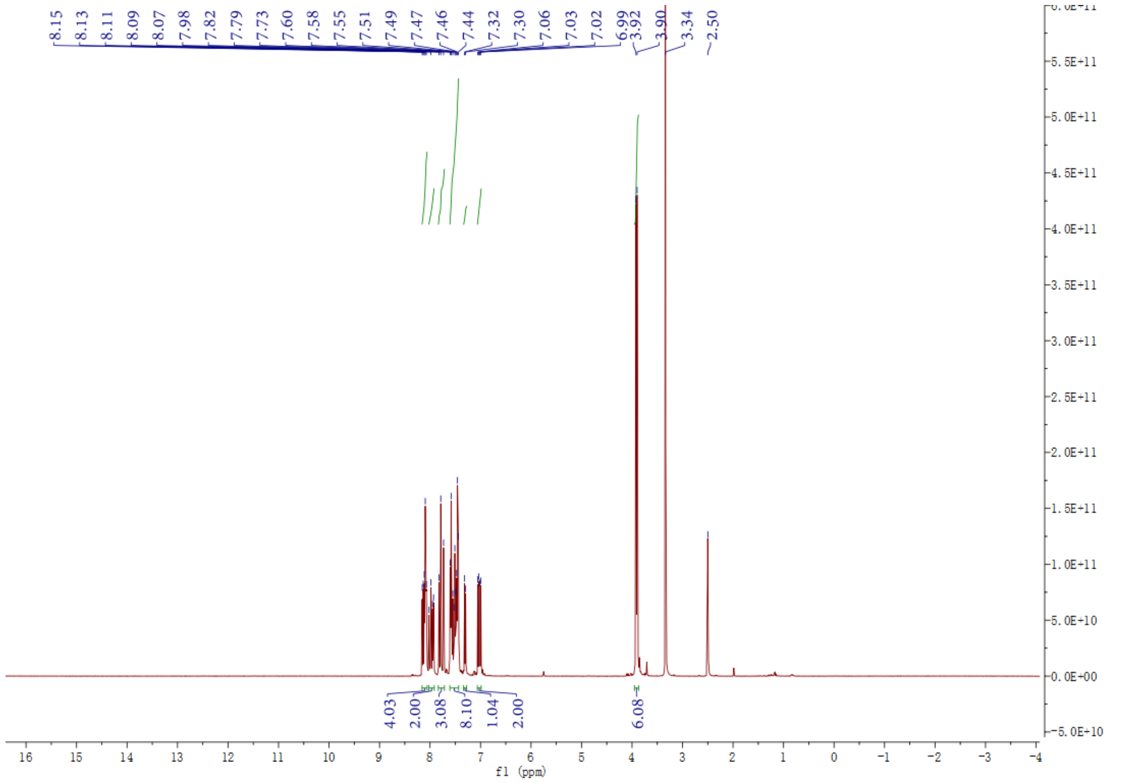

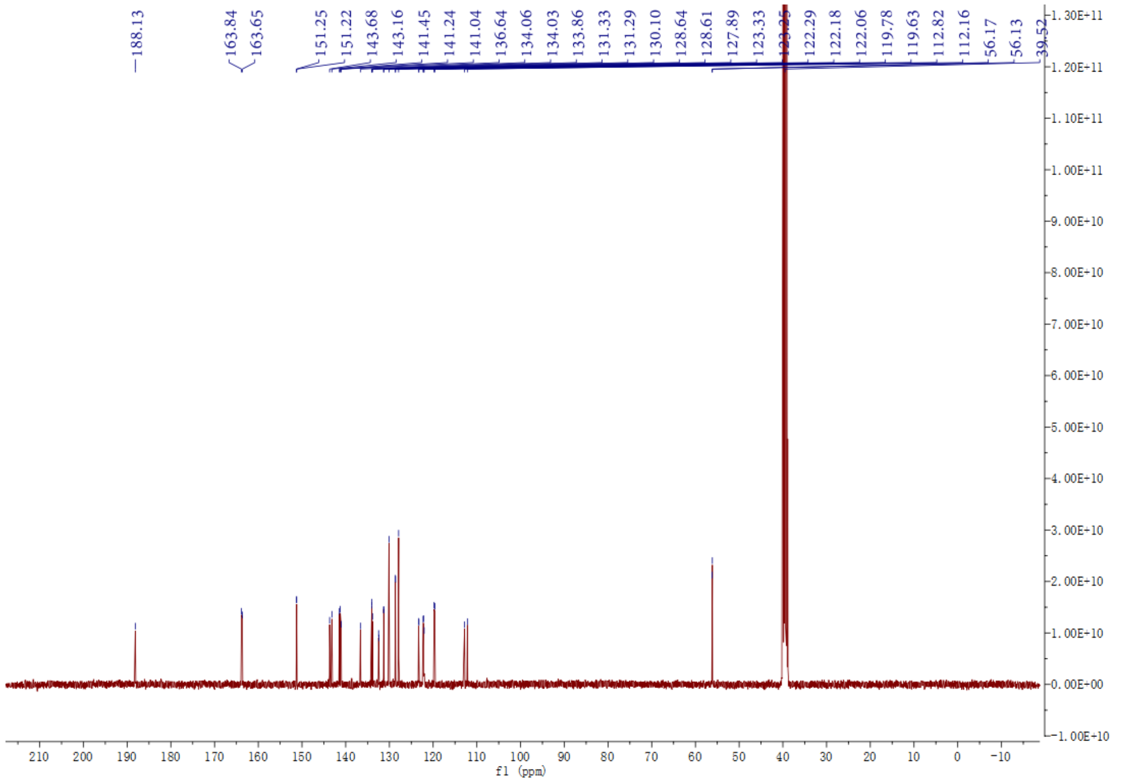

((E)-3-oxoprop-1-ene-1,3-diyl)bis(2-methoxy-4,1-phenylene) (2E,2'E)-bis(3-(3-chlorophenyl)acrylate) (**C3**)

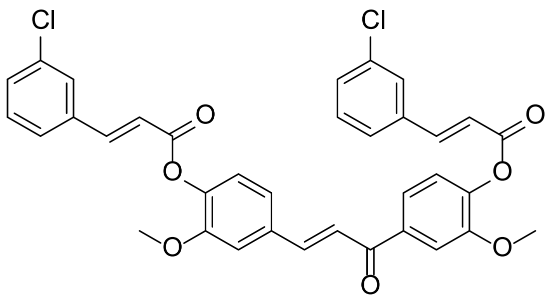
Cl
Cl
O
O
O
O
O
O
O

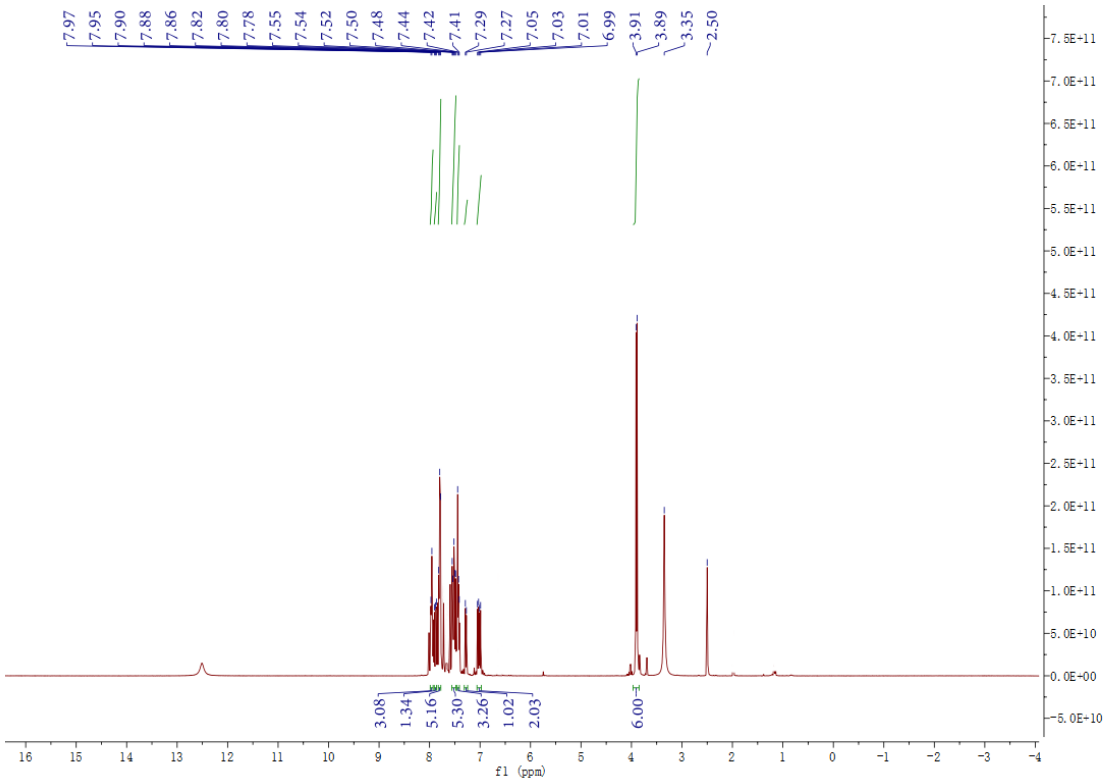
7.97
7.95
7.90
7.88
7.86
7.82
7.80
7.78
7.55
7.54
7.52
7.50
7.48
7.44
7.42
7.41
7.29
7.27
7.05
7.03
7.01
6.99
3.91
3.89
3.35
2.50
3.08
1.34
5.16
5.30
3.26
1.02
2.03
6.00
f1 (ppm)

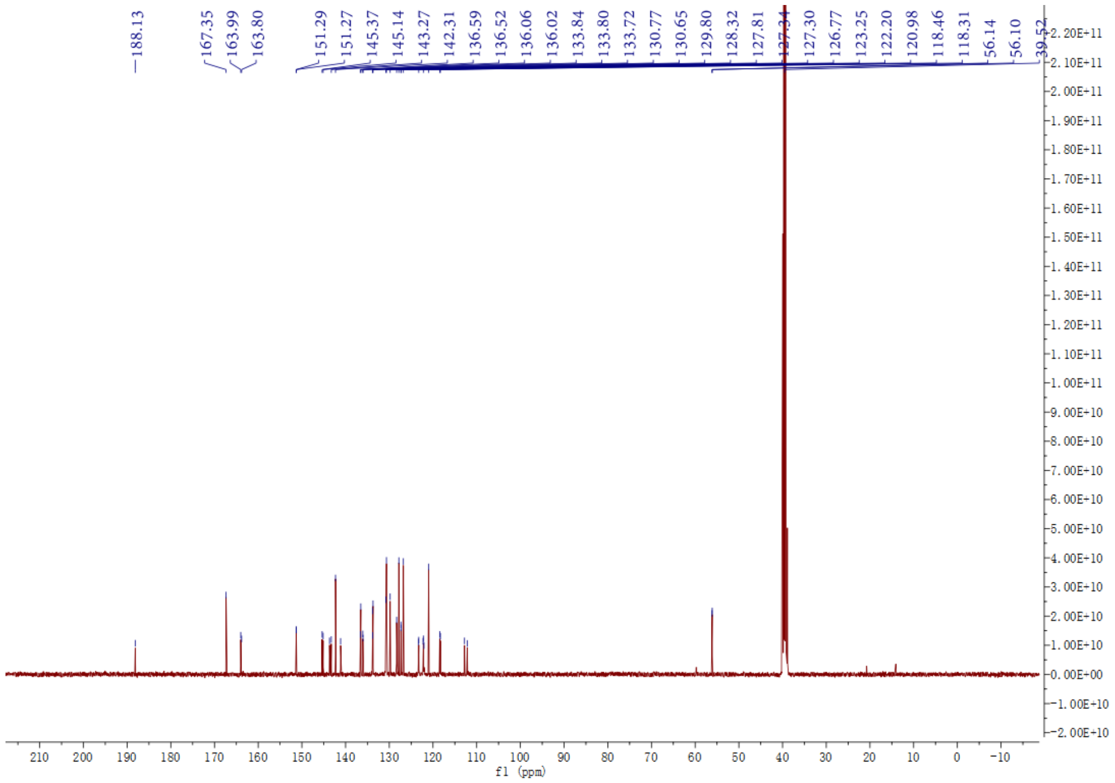

((E)-3-oxoprop-1-ene-1,3-diyl)bis(2-methoxy-4,1-phenylene) (2E,2'E)-bis(3-(3-(trifluoromethyl)phenyl)acrylate) (**C4**)

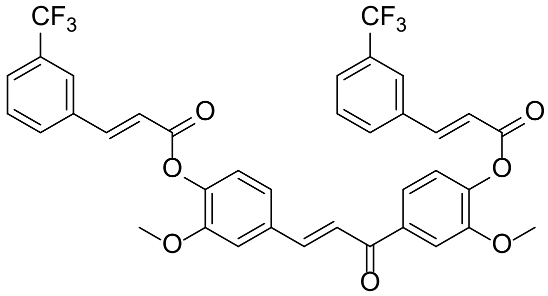

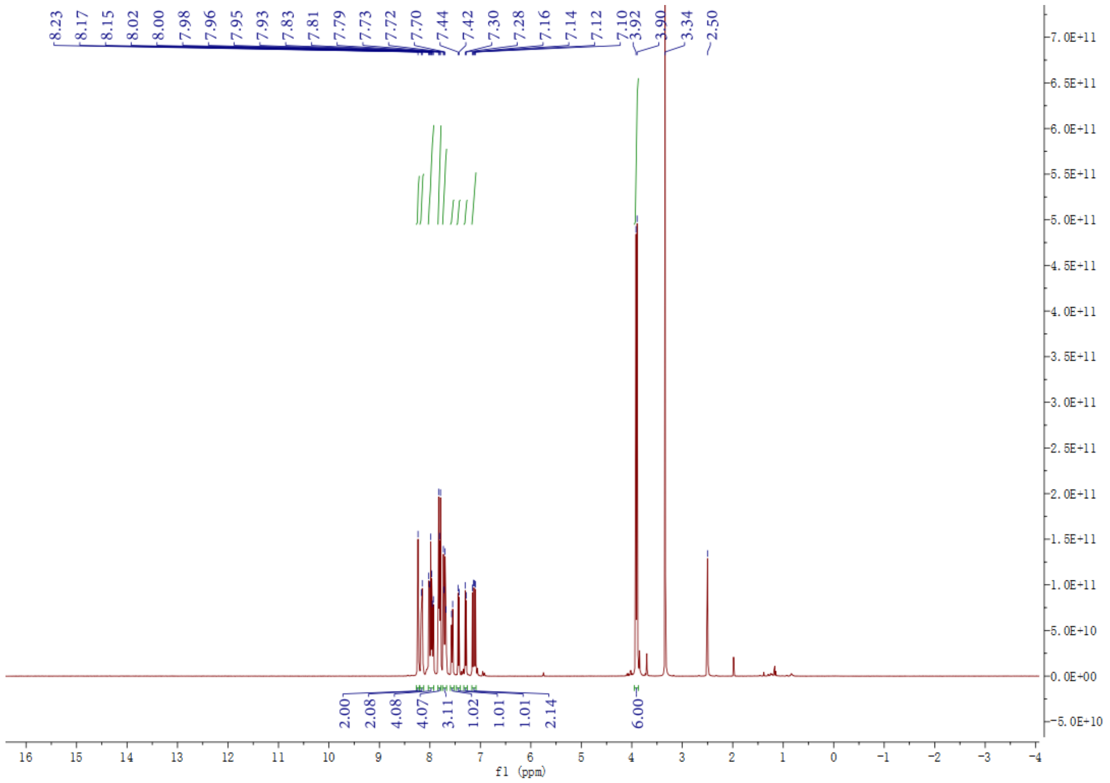

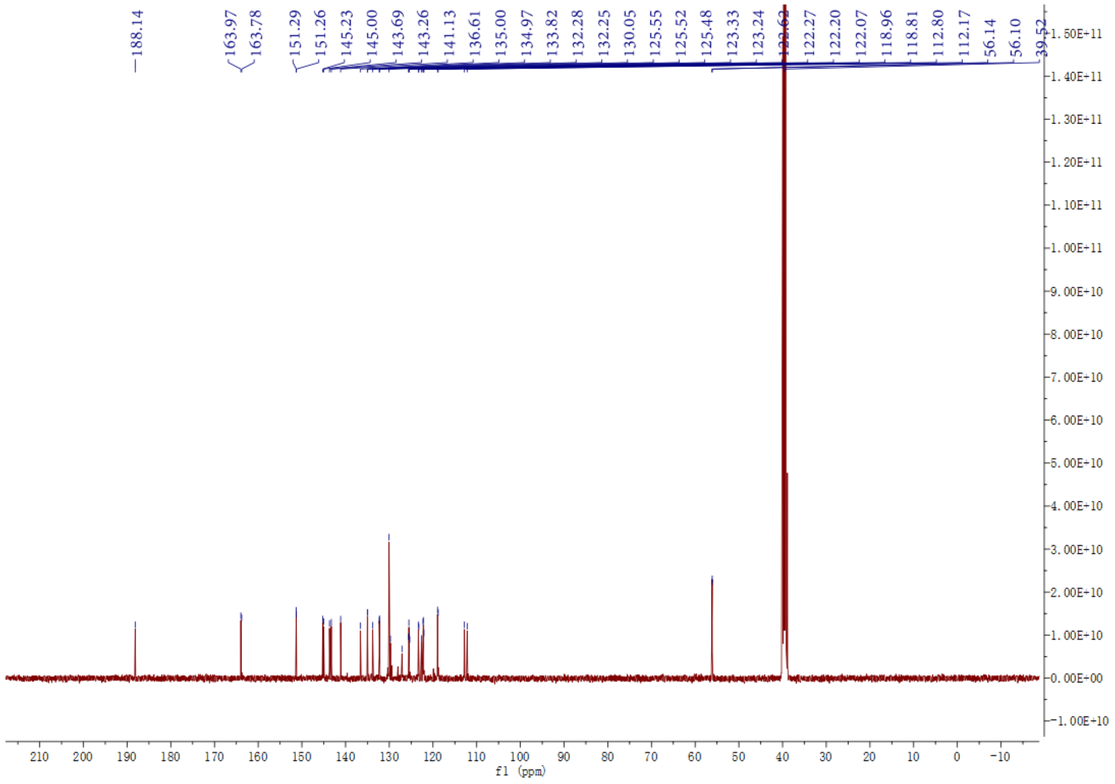

((E)-3-oxoprop-1-ene-1,3-diyl)bis(2-methoxy-4,1-phenylene) (2E,2'E)-bis(3-(4-(trifluoromethyl)phenyl)acrylate) (**C5**)

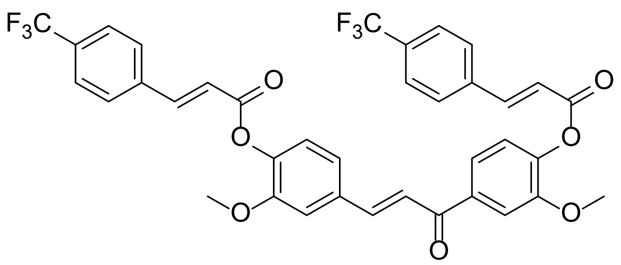
F3C
F3C
O
O
O
O
O
O
O

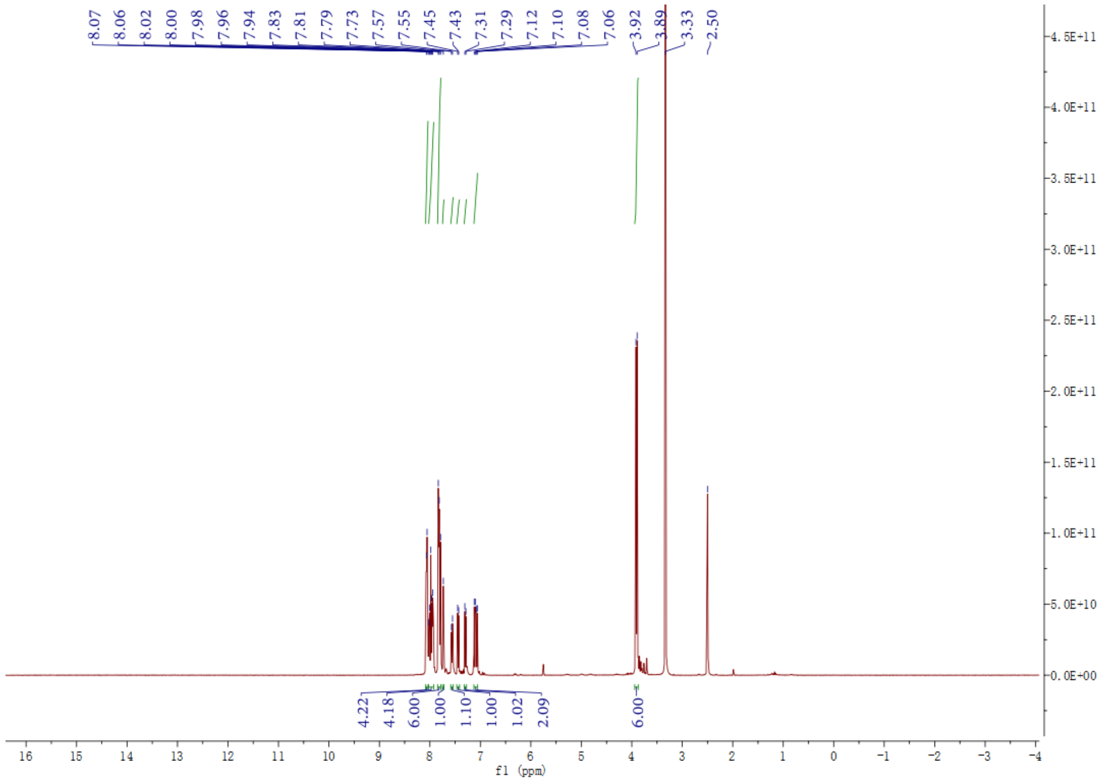
8.07
8.06
8.02
8.00
7.98
7.96
7.94
7.83
7.81
7.79
7.73
7.57
7.55
7.45
7.43
7.31
7.29
7.12
7.10
7.08
7.06
3.92
3.89
3.33
2.50
4.5E+11
4.0E+11
3.5E+11
3.0E+11
2.5E+11
2.0E+11
1.5E+11
1.0E+11
5.0E+10
0.0E+00
4.22
4.18
6.00
1.00
1.10
1.00
1.02
2.09
6.00
16 15 14 13 12 11 10 9 8 7 6 5 4 3 2 1 0 -1 -2 -3 -4
f1 (ppm)

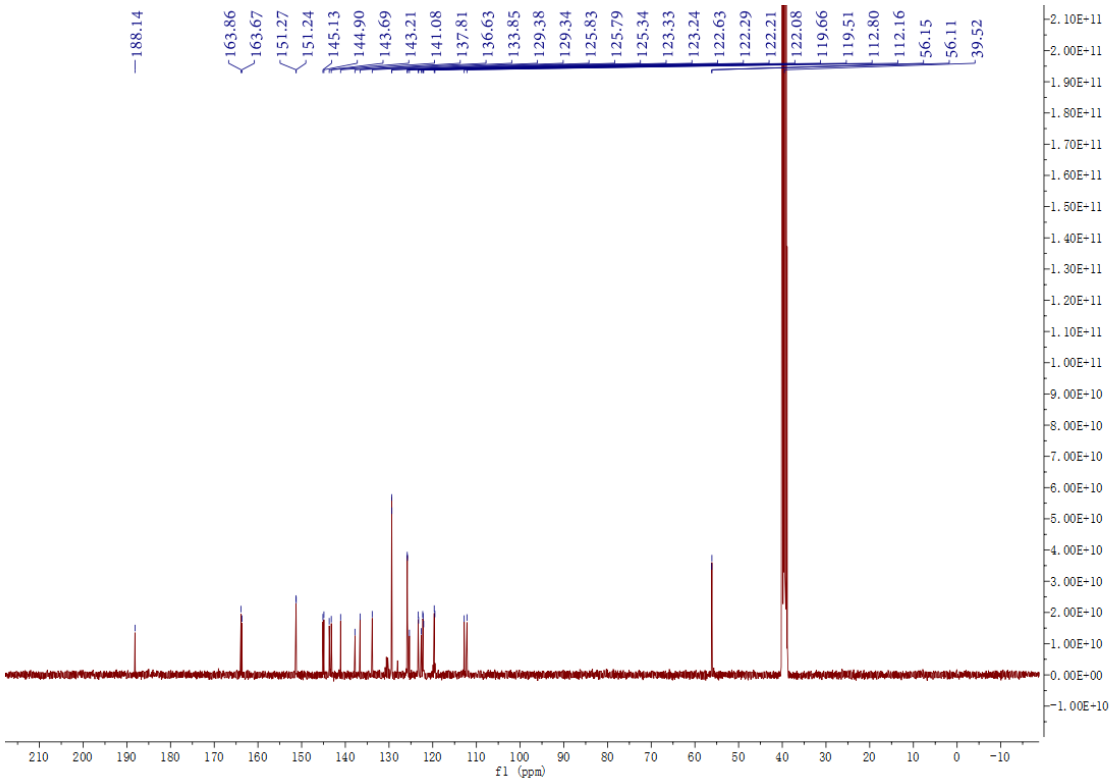

((E)-3-oxoprop-1-ene-1,3-diyl)bis(2-methoxy-4,1-phenylene) (2E,2'E)-bis(3-(p-tolyl)acrylate) (**C6**)

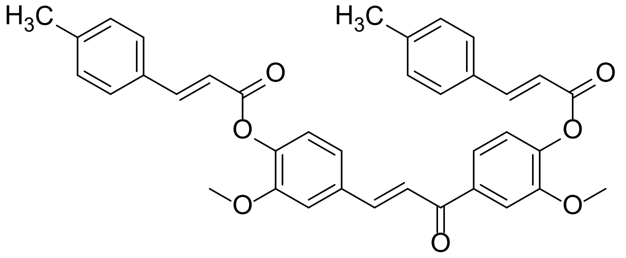

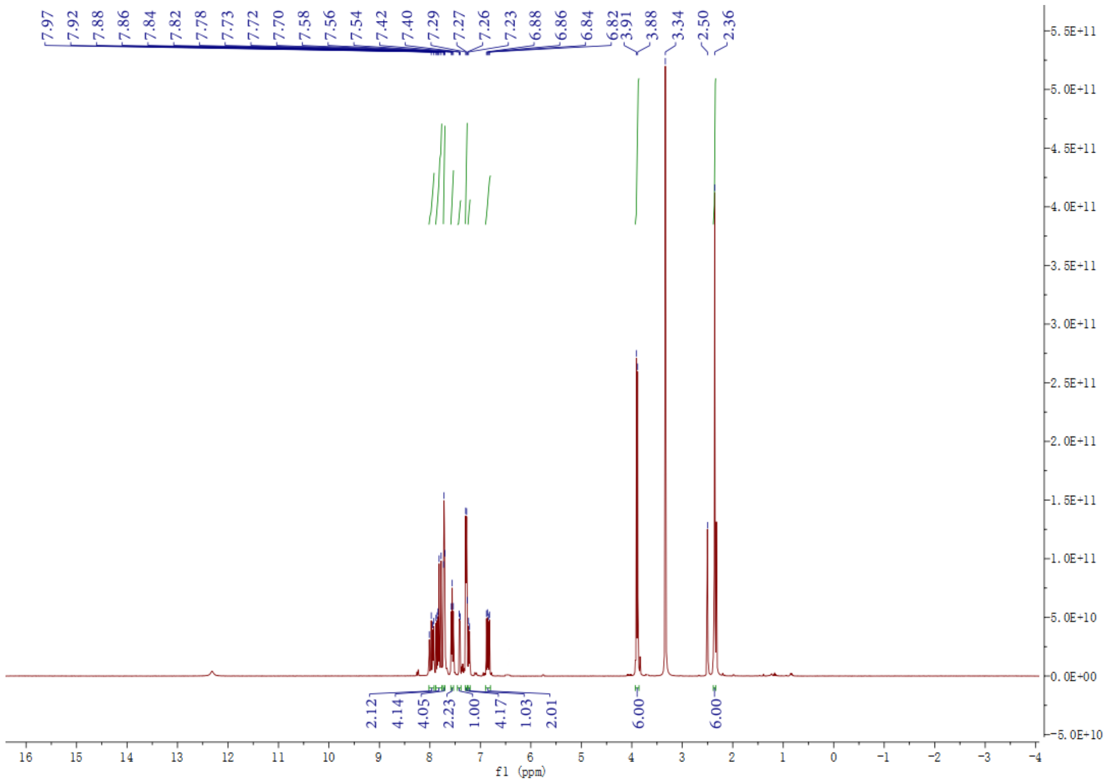
7.97
7.92
7.88
7.86
7.84
7.82
7.78
7.73
7.72
7.70
7.58
7.56
7.54
7.42
7.40
7.29
7.27
7.26
7.23
6.88
6.86
6.84
6.82
3.91
3.88
3.34
2.50
2.36
2.12
4.14
4.05
2.23
1.00
4.17
1.03
2.01
6.00
6.00
f1 (ppm)

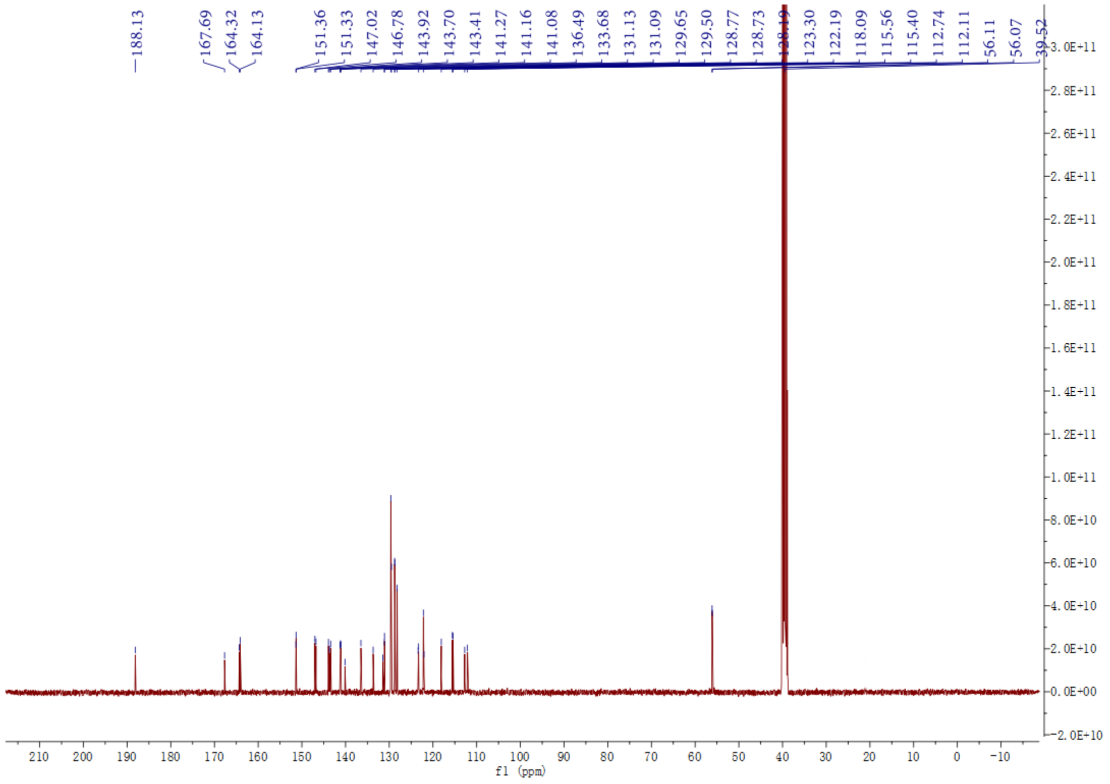
188.13
167.69
164.32
164.13
151.36
151.33
147.02
146.78
143.92
143.70
143.41
141.27
141.16
141.08
136.49
133.68
131.13
131.09
129.65
129.50
128.77
128.73
128.19
123.30
122.19
118.09
115.56
115.40
112.74
112.11
56.11
56.07
39.52
f1 (ppm)
